# Derivatives of Salarin A, Salarin C and Tulearin A—*Fascaplysinopsis* sp. Metabolites

**DOI:** 10.3390/md11114487

**Published:** 2013-11-11

**Authors:** Lee Goren Zur, Ashgan Bishara, Maurice Aknin, Drorit Neumann, Nathalie Ben-Califa, Yoel Kashman

**Affiliations:** 1 School of Chemistry, Tel Aviv University, Ramat Aviv 69978, Israel; E-Mails: goren5@gmail.com (L.G.Z.); ashganbi@gmail.com (A.B.); 2 Laboratorie de Chimie des Substances Naturelles et des Aliments, Faculté des Sciences et Techniques, Université de la Réunion, 15 Avenue Rene Cassin, BP 7151, Saint Denis 97715, Cedex 9, France; E-Mail: maurice.aknin@univ-reunion.fr; 3 Department of Cell and Developmental Biology, Sackler Faculty of Medicine, Tel Aviv University, Ramat Aviv 69978, Israel; E-Mails: histo6@post.tau.ac.il (D.N.); allouln@post.tau.ac.il (N.B.-C.)

**Keywords:** salarins, tulearins, *Fascaplysinopsis* sp., sponge, nitrogenous macrolide, leukemia cells

## Abstract

Derivatives of salarin A, salarin C and tulearin A, three new cytotoxic sponge derived nitrogenous macrolides, were prepared and bio-evaluated as inhibitors of K562 leukemia cells. Interesting preliminary SAR (structure activity relationship) information was obtained from the products. The most sensitive functionalities were the 16,17-vinyl epoxide in both salarins, the triacylamino group in salarin A and the oxazole in salarin C (less sensitive). Regioselectivity of reactions was also found for tulearin A.

## 1. Introduction

**Figure 1:**
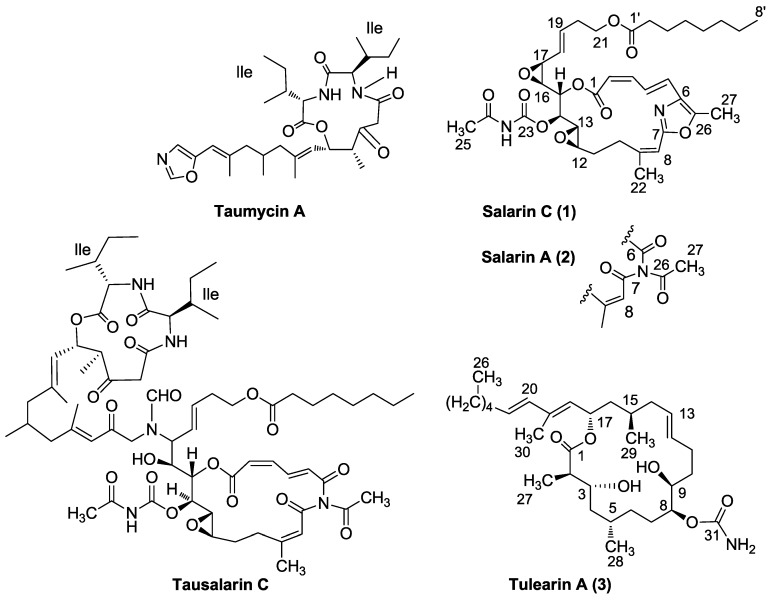
Representatives of *Fascaplysinopsis* sp. nitrogenous metabolites.

Four groups of nitrogenous macrolides (Figure 1) were isolated from the Madagascar *Fascaplysinopsis* sp. sponge collected in Salary Bay, ca. 100 km north of Tulear [1,2,3]. Among these metabolites the most abundant and active compound was salarin C (**1**), a potent inhibitor of proliferation of K562 leukemia cells (in concentration of 0.0005–0.5 μg/mL). The K562 cells underwent apoptotic death, as monitored by cell cycle analysis, annexin V/propidium iodine staining and caspase 3 and caspase 9 cleavage [1,2,3].

Interestingly, a remarkable change in the amounts of the different compounds was observed in various collections of the sponge. This, together with the resemblance of the functional groups to functionalities found in microorganism metabolites [1,2,3,4,5,6,7], suggested that the four groups originate from guest microorganisms rather than from the sponge itself.

The salarins contain seven functional groups which complicate the chemistry of these compounds. Among the currently identified ten naturally occurring salarins (A–J) the most active one is salarin C (**1**) which changes under light and air to salarin A (**2**) [2,8]. The suggested mechanism for this transformation is a singlet oxygen cleavage of the oxazole via a cycloaddition adduct [9,10]. The latter change, which occasionally occurs while performing chemical reactions with salarin C, complicated the chemistry even more. It was therefore advisable to compare reactions of salarin C with those of salarin A, *vide infra*. Among the more reactive moieties of **1** is the 16,17-vinyl epoxide which, as expected, is more reactive than the 12,13-epoxide, thus enabling regioselective reactions. Different openings of the vinyl epoxide were undertaken for: (a) A preparation of α-methoxy-α-trifluoromethylphenylacetic acid (MTPA) derivatives for determination of the absolute stereochemistry of **1**; (b) Linking a spacer for immobilization of the molecule; (c) Preparation of more polar derivatives of **1** for SAR studies; and (d) Supporting the suggested biogenesis of tausalarin C (Figure 1) [4].

In five of the natural salarins the 16,17-epoxide is replaced by the corresponding vicinal 16,17- or 16,19-diol obtained, most likely, due to an allylic rearrangement.

## 2. Results and Discussion

**Scheme 1:**
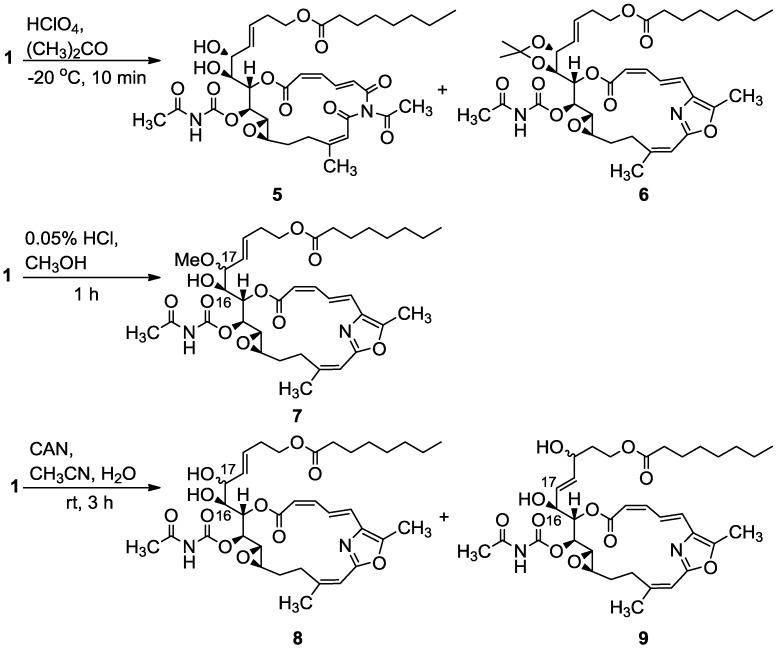
Acid catalyzed openings of the vinyl epoxide of salarin C (**1**).

Selective acid catalyzed openings of the vinyl epoxide were disclosed with catalytic amounts of HClO_4_ in acetone or HCl in MeOH. The former provided the major compounds 16,17-diol **5** and its acetonide derivative **6**, and the latter gave the 16-hydroxy-17-methoxy derivative **7** (Scheme 1). Other tested conditions afforded complex mixtures. According to the NMR data of the dieneoate moiety, which is very sensitive to the stereochemistry of the double bonds (e.g., the 8.31 ppm chemical shift of H-4) it was evident that this functional site stayed intact [11]. The same was the case with other moieties that did not take part in the reaction whose NMR data remained unchanged (for all intact sites Δδ_H_ ± 0.1, Δδ_C_ ± 0.5, the same is the case for the other derivatives) [11].

Characteristic in the proton NMR spectra of compounds **5**–**7** were the disappearance of the 16,17-epoxide protons around 3 ppm and the appearance of methinoxy proton signals around 4 ppm (see Experimental and Supporting Information). All above reactions were very sensitive to the conditions and afforded low yields. It can be assumed that C-16 in compounds **5**–**7** maintains its (16R*) stereochemistry (protonation of the epoxide followed by opening of the O–C17 bond), which is doubtful for C-17, as the intermediate allylic C-17 carbocation can give two epimers due to a nucleophilic attack from both sides of the molecule and/or allylic rearrangement as shown, for e.g., in **9** (Scheme 1).

The 17 methoxy location was determined from COSY (H-15 to -18) and HMBC data from the methoxyl to C-17. A 16,17-*threo* configuration was deduced for acetonide **6** from the very close chemical shifts of the two acetonide methyl groups (28.7q and 1.44s for Me-29, 27.3q and 1.43s for Me-30, and 101.7s for C-28 of the acetonide), as well as the *J*_H16,H17_ = 7.8 Hz coupling constant [12], thus determining the 17S*-stereochemistry for both **5** and **6**.

Treating salarin C with CAN (cerium ammonium nitrate) in acetonitrile (Scheme 1), a reaction suggested for opening radically vinyl epoxides to the corresponding diols [13], provided diol **8** and the allylic rearranged diol **9**. As CAN is highly acidic it is not clear if the reagent or the accompanying HNO_3_ acid are responsible for the epoxide opening (Scheme 1). A single diastereoisomer was isolated; this was also the case with the other epoxide openings.

**Scheme 2:**
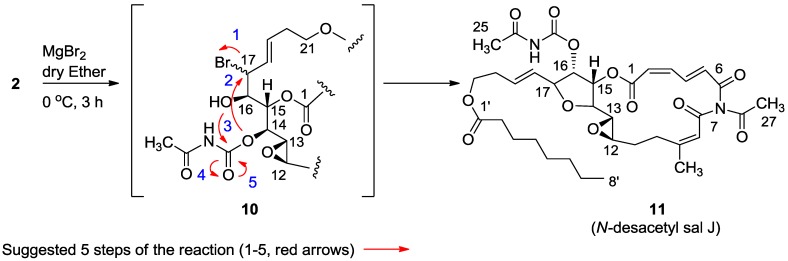
Mild Lewis acid opening of the vinyl epoxide with MgBr_2_ in ether, via intermediate **10**.

**Figure 2:**
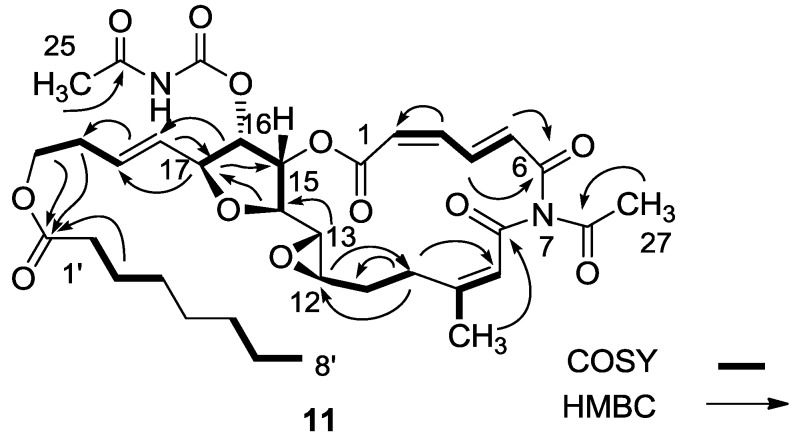
Selected 2D-correlations of compound **11**.

Another interesting opening of the 16,17-epoxide was with the freshly prepared Lewis acid MgBr_2_ in ether, conditions known to afford from vinyl epoxides the corresponding bromohydrins (Scheme 2) [14]. In the event, the expected bromohydrin (**10**) was initially indeed obtained as inferred from the MS. However, the compound was not stable and rearranged overnight to a stereoisomer of *N*-desacetyl salarin J (**11**) as concluded from the NMR data [6]. Namely, H-16 moved to 5.04 ppm (dd, *J* = 7.0, 5.1 Hz) and H-17 to 4.40 ppm (t, *J* = 6.8 Hz). C-16 was found to resonate at 75.4 and C-17 at 80.9 ppm. The chemical shifts of the intact moieties did not change. The main 2D correlations are depicted in Figure 2. A suggested mechanism for the rearrangement is given in Scheme 2. Because of the small *J* values in the THF ring, the stereochemistry around the ring could not be determined.

**Scheme 3:**
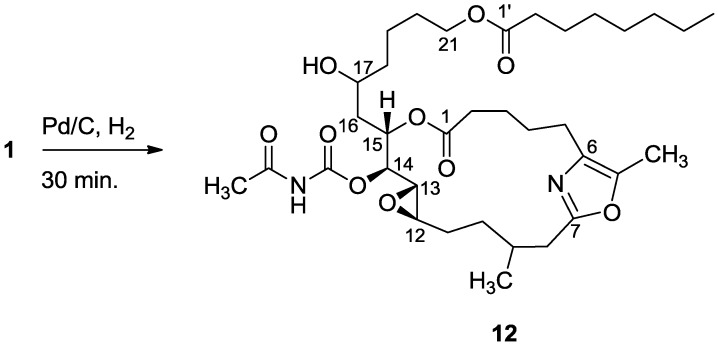
Hydrogenation of salarin C.

**Figure 3:**
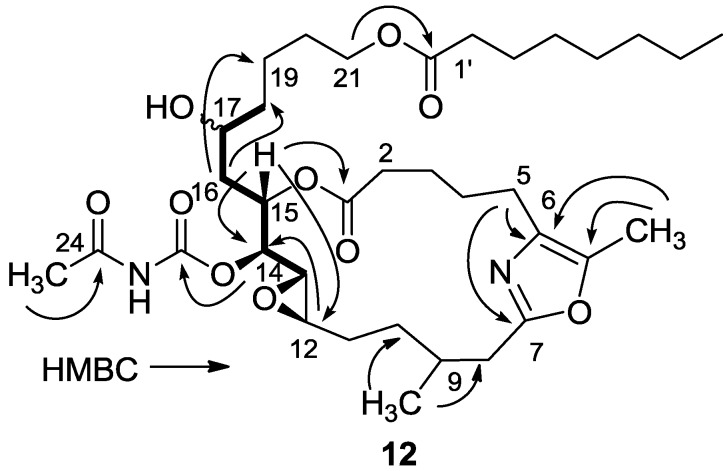
Selected HMBC correlations for **12**.

Worth mentioning is another opening of the 16,17-epoxide, namely, hydrogenation of salarin C which saturated the four double bonds, without affecting the oxazole, and opened up the 16,17-vinyl epoxide to the 17-alcohol (**12**, Scheme 3 and Figure 3). The 17-hydroxy location is suggested on the basis of COSY correlations from H-13 to H_2_-18 (see Experimental Section) and supported by HMBC correlations (Figure 3). Furthermore, the *trans* *J*_12,13_ = 2.2 Hz value confirmed that the 12,13-epoxide remained intact. Therefore, the configuration of C-17 could not be determined.

**Scheme 4:**
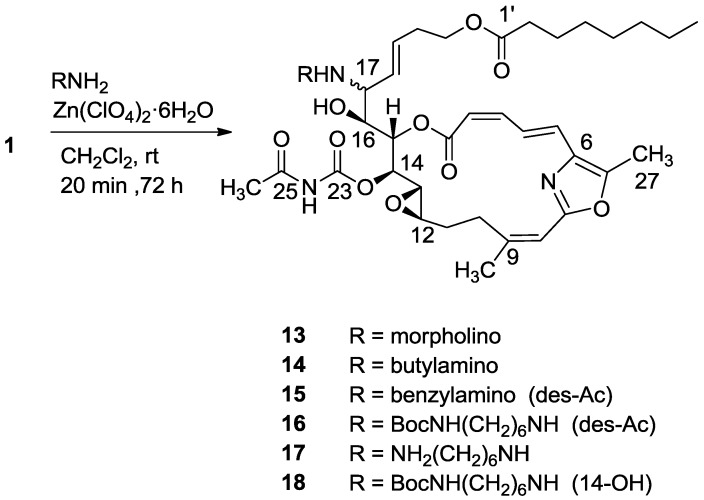
Reactions of salarin C with different amines in the presence of Zn(ClO_4_)_2_·6H_2_O.

**Scheme 5:**
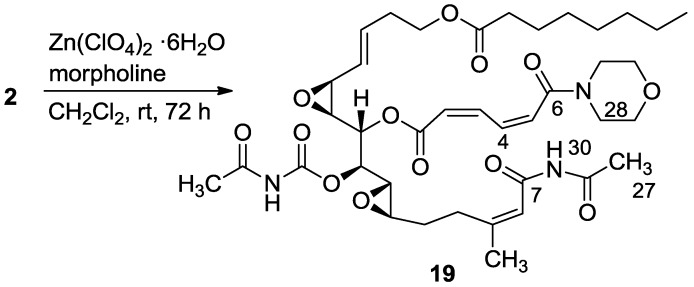
Reaction of salarin A with morpholine in the presence of zinc perchlorate.

The next studied reaction was an amine/Zn(ClO_4_)·6H_2_O opening of the 16,17-vinyl epoxide [15], which provided, as expected, the 16-hydroxy-17-amino-derivatives (**13**–**18**) (Scheme 4) [16]. The reaction was performed both on salarin C and salarin A. Unexpectedly, the two compounds behaved differently. While salarin C gave the expected 16-hydroxy-17-amino derivatives, via *infra*, (**13**–**18**), in the case of salarin A the epoxide remained intact while the triacyl moiety of the macrocycle of **2** (compound **19**) was found to be more reactive and opened up as depicted in Scheme 5. The only changed site in the NMR was that of CH-16 and CH-17; e.g., for compound **14**, H-16 moved to 3.60 ppm (t, *J* = 7.7 Hz), H-17 to 2.83 ppm (t, *J* = 7.7 Hz), C-16 resonated at 71.6 ppm and C-17 at 64.8 ppm.

**Figure 4:**
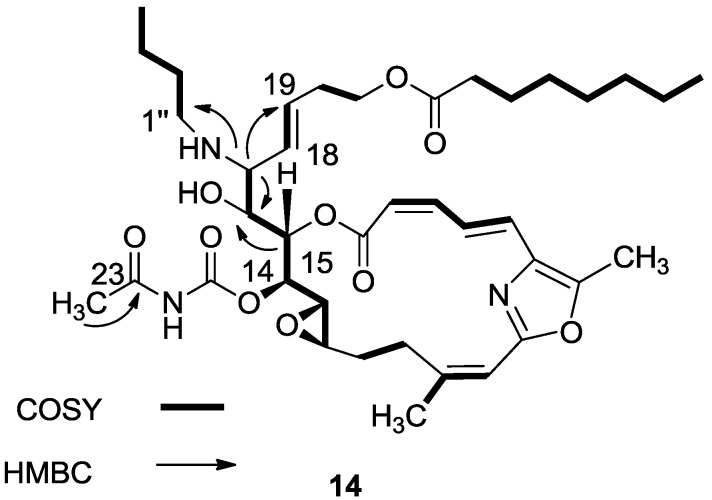
Selected 2D-correlations of compound **14**.

HMBC and selected COSY correlations for hydroxyl amine **14** are depicted in Figure 4 and similar 2D correlations were seen for **13** and **17**. Compounds **15** and **16** lost the *N*-acetyl group. For compound **18**, the acetyl carbamate was absent as seen in the ^13^C and ^1^H-NMR spectra (Figure 4).

Carbonyls C-6 and C-7 of compound **19** moved up-field in the NMR spectrum from 171.9 to 164.0 and from 167.9 to 163.6, respectively. Evidence for change in the molecule was further obtained by FABMS *m*/*z* 796.1, (C_39_H_55_N_3_O_13_Na, M + Na^+^).

**Figure 5:**
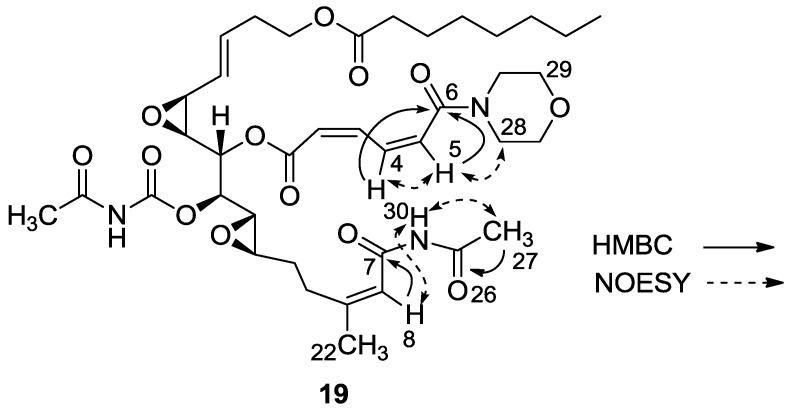
2D-NMR key correlations for the changed site of compound **19**.

Observed NOEs between H-4 and H-5, between H-5 and H-28, and between NH-30 and H-8, as well as HMBC correlations from NH-30 to C-27, and from H-4 to C-6 differentiated between three possible cleavages of the triacylamine moiety (*i.e.*, C-6/C-7; C-30/C-6 or C-30/C-7) determining the structure of **19** (Figure 5).

In addition to preliminary SAR studies of salarin C derivatives, the amination of salarin C was also directed to obtain a congener possessing a spacer for immobilization of salarin C (compounds **17** and **18**, Scheme 4). However, as compounds **17** and **18** already lost activity, no further reactions were undertaken. Also, opening of the vinyl epoxide with an amine supported the earlier suggested biogenesis of tausalarin C, *i.e.*, coupling between salarin A (or C) and pre-taumycin that, by a different route, affords taumycin A [4].

**Scheme 6:**
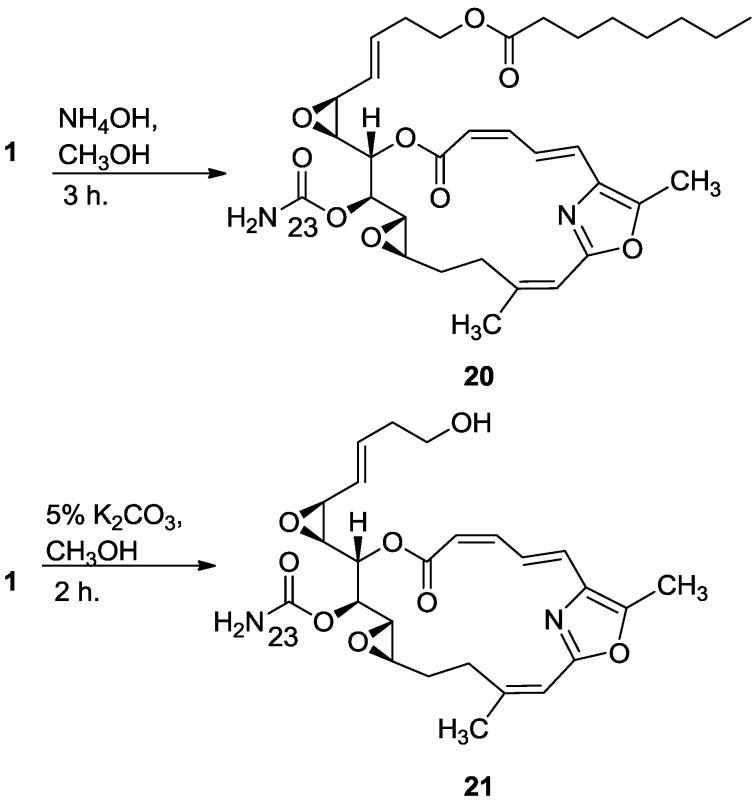
Treatment of salarin C with base.

Next the preparation of a more polar derivative of salarin C was undertaken to try to improve its solubility in water. As was demonstrated in acidic conditions, salarins were also found highly sensitive in basic conditions. Short treatment of salarin C with different bases first gave the *N-*desacetyl derivative **20** (Scheme 6) characterized by the disappearance of the acetyl resonances in the ^1^H-NMR spectrum, while prolonging the reaction (2 h), using a stronger base, also led to hydrolysis of the octanoate ester (**21**, Scheme 6). Further elongation of the hydrolysis resulted in an unidentified complex mixture.

**Scheme 7:**
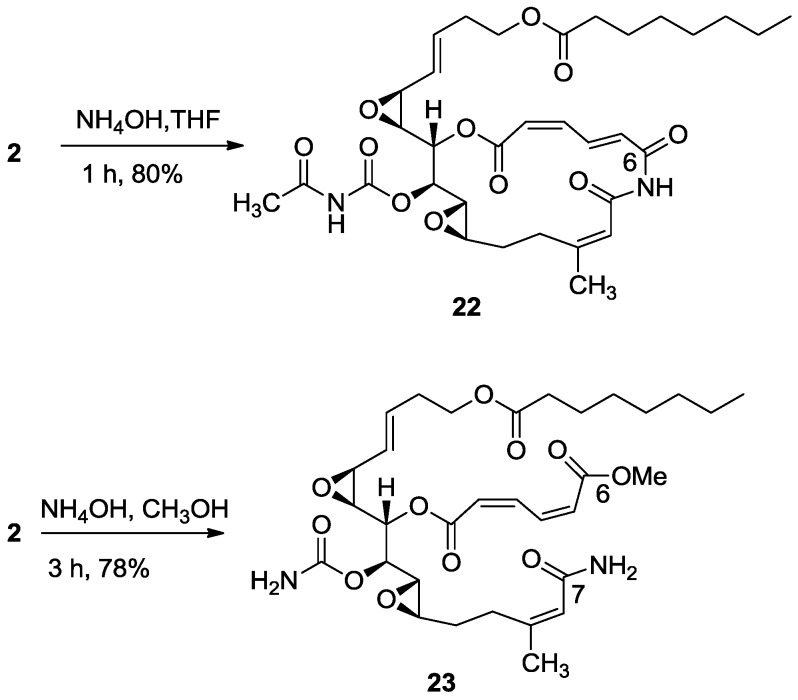
Treatment of salarin A with base.

As shown above, in different reactions, salarin A behaved differently from salarin C. The same was the case with the hydrolysis. Again, in the first hydrolysis step the *N-*acetyl group split off to give compound **22**, known naturally as salarin E (most likely together with methyl acetate which was not isolated) [6], while the second step was the opening of the azamacrocycle, providing the C-6 methyl ester–C-7 amide (**23**, Scheme 7).

The ^1^H-NMR spectrum of **23** showed a three proton singlet at 3.78 ppm, indicating the presence of a methyl ester, accompanied by the disappearance of the two acetates (C-25 and C-27 of **2**).

**Figure 6:**
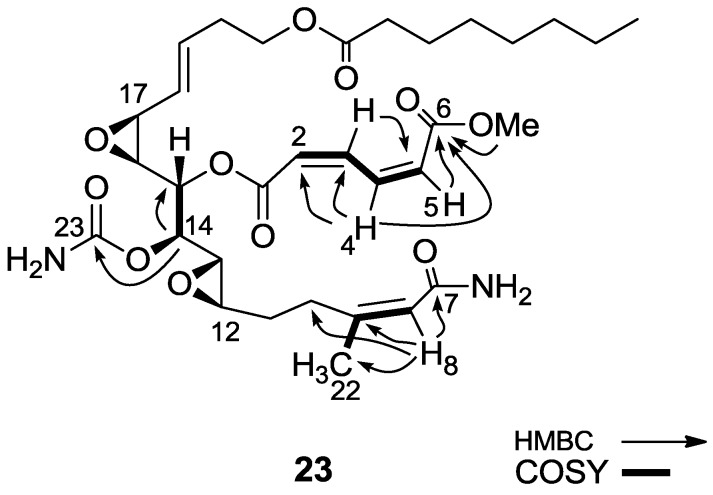
Selected 2D NMR correlations for the modified site of compound **23**.

Two structures are possible for **23**, *i.e.*, in one C-6 is the ester and C-7 the amide, or *vice versa*. HMBC correlations from H-4, H-5 and the methoxyl to carbonyl C-6 and from H-8 to CH_3_-22 and to the amide carbonyl C-7 clarified the structure (**23**, Scheme 7); key CH-correlations are depicted in Figure 6.

**Scheme 8:**
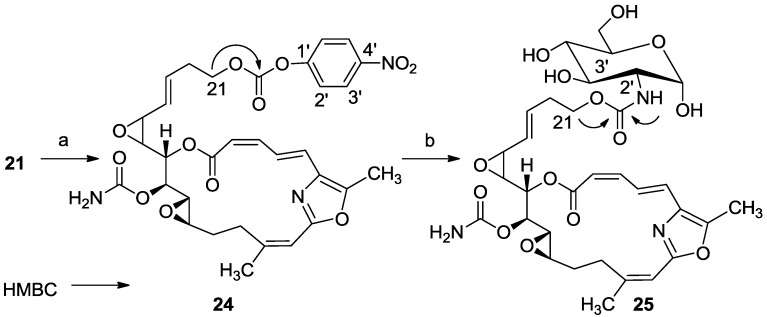
Synthesis of salarin-aminosugar **25**. Reagents and conditions: (**a**) bis(4-nitrophenyl) carbonate, Et_3_N, DMAP, CH_2_Cl_2_, 2 h; (**b**) glucosamine, Et_3_N, dry DMF, 2 h, rt.

After obtaining compound **21**, its attachment to glucosamine (Scheme 8) was achieved via activation of the primary C(21)H_2_OH group through the *p*-nitrophenyl carbonate group (**24**), which was then converted to the sugar carbamate **25** (Scheme 8).

The HRESI-MS and NMR data of **23** confirmed its structure. Downfield shifts of H-2′ of the glucosamine (∆δ_H_ = +0.9 ppm), compared to the starting free glucosamine (δ_H_ 2.67 m), and also of methylene H_2_-21 (δ_H_ 4.12 m) and HN-2′ (δ_H_ 5.16 s), as well as ^3^*J*_CH_ correlations to the carbamate carbonyl (δ_C_ 155.9 s), evidenced the connection of the sugar **21**.

Tulearin A (**3**) belongs to the second group of the *Fascaplysinopsis* sp. metabolites (tulearins A–C) [5] their cytotoxicity to K562 leukemia cells was lower than that of salarin C (72 h, 1 μM, ~60% inhibition of proliferation), while salarin C inhibited completely in this concentration [3,5]. However, it was decided to check the bioactivity of these derivatives.

**Figure 7:**
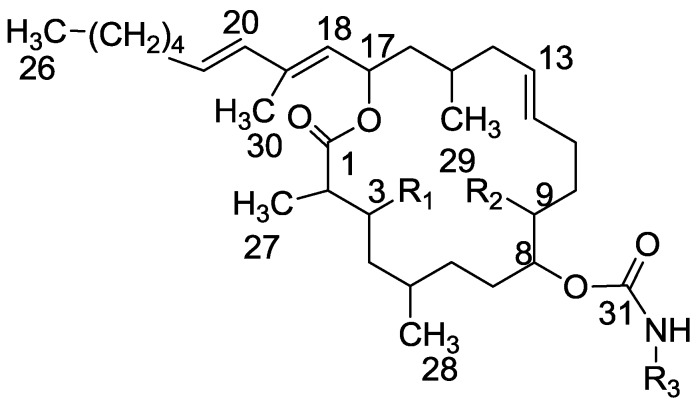
Tulearin A and modification positions (Tulearin A: R_1_,R_2_ = OH, R_3_ = H).

In our previous report, Diels Alder reactions of the 18,20-diene of tulearin A, as well as a modification of the 8-carbamate to a crystalline carbonate, were reported [5]. For the absolute configuration determination of the tulearins we prepared regioselectively the 9-MTPA esters to apply the modified Mosher method [17]. The latter 9-ester was obtained exclusively without the 3-isomer [5]. Thus, it was interesting to find out where the other esterifications would take place. Acetylation, mesylation, tosylation, benzoylation and *p*-bromobenzoylation were performed. All first gave the 9-mono derivative (**26**–**34**), followed by a slower esterification of the 3-hydroxyl. The 9-hydroxyl esterification location became clear from the H-9 chemical shift to a lower field (Figure 7 and Experimental Section).

The only exception was the 3-carbonate obtained from the reaction of **3** with *p*-nitrophenyl chloroformate (PNPCl, **35**, Table 1). To exclude/diminish the possibility that an initially obtained 9-isomer rearranges to the 3-isomer via a 3,9-bridge, reactions with oxalyl chloride and thionyl chloride were undertaken—no transannular bridge could be revealed.

In addition, the acylation of the carbamate was examined (**33** and **34**, Table 1).

**Table 1:** Tulearin A derivatives.

	Product	R_1_	R_2_, R_3_	Yield(%)
**26**	Anhyrous Pyr, TsCl 0 °C, rt, 48 h	OTs	OTs	20%
**27**		OH	OTs	35%
**28**	Et_3_N, p-bromobenzoylchloride, CH_2_Cl_2_, rt, 24 h	OH	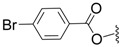	90%
**29**	MsCl, Pyr 0 °C, rt, 3 h	OMs	OMs	61%
**30**	Benzoyl chloride, Et_3_N, CH_2_Cl_2_, rt, 24 h	OH	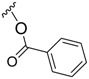	40%
**31**	Ac_2_O, Pyr, rt, 0.5 h	OH	OAc	60%
**32**	Ac_2_O, Pyr, rt, 48 h	OAc	OAc	90%
**33**	Ac_2_O, Pyr, DMAP (catalitic) rt, 7 days	OAc	R_2_ = OAc R_3_ = Ac	20%
**34**	Hexanoyl chloride, DMAP (catalitic), Et_3_N, CH_2_Cl_2_, rt, 48 h	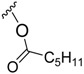	R_2_,R_3_ = 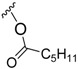	30%
**35**	PNPCl, DMAP, CH_2_Cl_2_, rt, 48 h	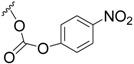	OH	35%

**Scheme 9:**
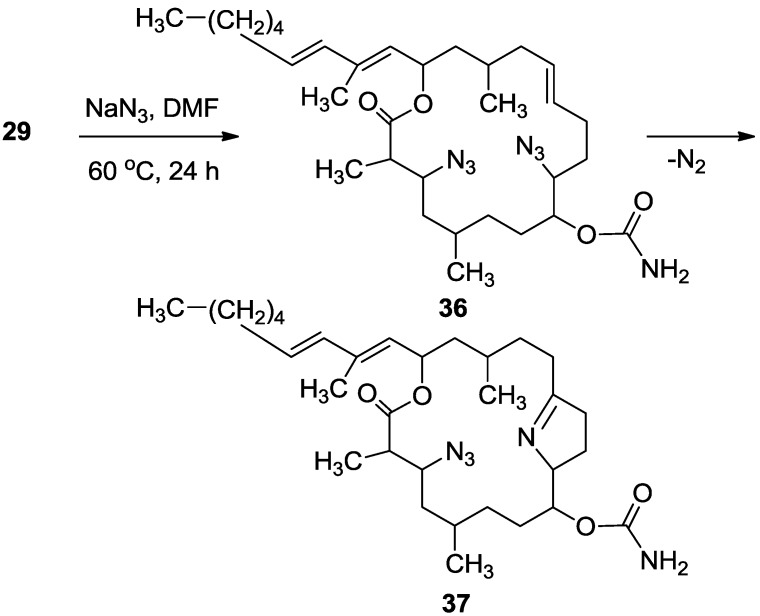
Change of dimesyl **29** to **37** via **36**.

Next, dimesyl **29** was transformed to the 3,9-diazido derivative (**36**) which was found, in low yields, to slowly transfer to the cycloadditon pyrolidine product **37** (Scheme 9) [18]. HRESI-MS established the formula C_31_H_52_N_5_O_4_, found *m*/*z* 558.4011 (MH^+^; calculated 558.4019).

**Figure 8:**
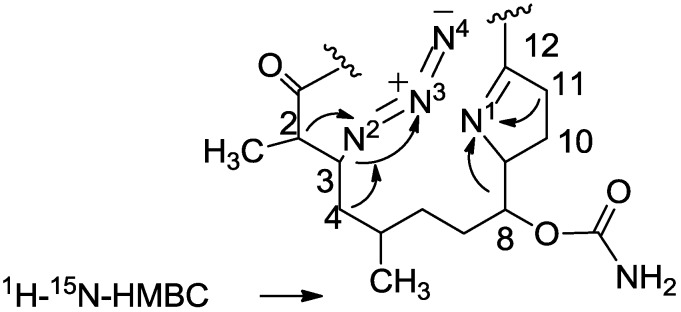
^1^H-^15^N-HMBC correlations for compound **37**.

The ^15^N-chemical shifts of the nitrogen atoms of three out of the five molecules of **37** were established by a ^1^H-^15^N-HMBC experiment, namely, correlations from H-2 and H-4 to N^2^ at 72 ppm, from H-3 to N^3^ at 243 ppm, and from H-8 and H-11 to N^1^ at 310 ppm. No coupling was observed between H-3 and N^4^, most likely because of too small a polarization transfer, thus the carbamate nitrogen was not disclosed (Figure 8) [18]. Furthermore, C-12 resonances, were as expected from an imine at δ_C_ 179.2 ppm, CH-3 at δ_C_ 61.8 and δ_H_ 3.78 ppm, and CH-9 at δ_C_ 74.8 and δ_H_ 4.20 ppm.

## 3. Experimental Section

### 3.1. Extraction and Isolation [1,2,3,4,5,6,7]

For general methods, see Supporting Information.

### 3.2. Salarin Derivatives

The NMR data of the salarin derivatives is given only for the transformed sites, for the rest of the functional moieties of the molecules the chemical shift changes of the various atoms were minimal (Δδ_H_ ± 0.1, Δδ_C_ ± 0.5). Thus for example the chemical shift of H-4 is very characteristic for the dienoate moiety and its surroundings (8.31 ppm). Full representative NMR spectra are given in the Supporting Information. The NMR data of the changed sites are given for clarity in order of the atom numbers.

#### 3.2.1. Compounds **5** and **6**

To a mixture of salarin C (20 mg, 0.03 mmol) in acetone (5 mL), was added HClO_4_ (7%, 0.1 mL) at −20 °C and the reaction was stirred for 10 min. The reaction mixture was neutralized with aqueous NaHCO_3_ solution, evaporated and the residue diluted with water and extracted with DCM. The combined organic extract was dried over anhydrous MgSO_4_ and evaporated. The residue was purified by VLC (vacuum liquid chromatography, petroleum ether/ethyl acetate, 8:2) to afford **6**, as a colorless oil, 3 mg (20%) and **5**, a second colorless oil, 4.5 mg (21%). **6**: [α]_D_^23^ +22 (*c* 0.1, CHCl_3_); NMR data for modified site (C14–C19): ^1^H-NMR (CDCl_3_, 400 MHz) δ 4.80 (dd, *J* = 8.7, 2.2 Hz, 1H, H-14), 5.61 (m, 1H, H-15), 4.00 (dd, *J* = 9.8, 7.8 Hz, 1H, H-16), 4.27 (t, *J* = 7.8 Hz, 1H, H-17), 5.41 (dd, *J* = 15.6, 8.1 Hz, 1H, H-18), 5.64 (m, 1H, H-19); acetonide moiety: 1.44 (s, 3H, H-29), 1.43 (s, 3H, H-30); ^13^C-NMR (CDCl_3_, 100 MHz) δ 76.4 d (C-14), 72.3 d (C-15), 78.3 d (C-16), 82.4 d (C-17), 127.5 (C-18), 131.8 d (C-19); acetonide moiety: 101.7 s (C-28), 28.7 q (Me-29), 27.3 q (Me-30). FABMS *m*/*z* 735.3, (C_38_H_52_N_2_O_11_Na, M + Na^+^); **5**: [α]_D_^23^ +72 (*c* 0.05, CHCl_3_); NMR data for modified site (C14–C19): ^1^H-NMR (CDCl_3_, 400 MHz) δ 5.04 (t, *J* = 4.3 Hz, 1H, H-14), 5.44 (dd, *J* = 6.8, 4.3 Hz, 1H, H-15), 3.94 (t, *J* = 6.8 Hz, 1H, H-16), 4.37 (t, *J* = 7.9 Hz, 1H, H-17), 5.48 (dd, *J* = 15.4, 7.9 Hz, 1H, H-18), 5.77 (dt, *J* = 15.4, 7.9 Hz, 1H, H-19); ^13^C-NMR (CDCl_3_, 100 MHz) δ 73.1 d (C-14), 73.4 d (C-15), 78.3 d (C-16), 80.9 d (C-17), 128.3 d (C-18), 133.0 d (C-19). FABMS *m*/*z* 727.3, (C_35_H_48_N_2_O_13_Na, M + Na^+^).

#### 3.2.2. Compound **7**

A solution of salarin C (12 mg, 0.02 mmol) in MeOH (10 mL) was treated with methanolic HCl solution (0.05% v/v, 0.1 mL). The mixture was stirred at rt in the dark for 1 h. The reaction mixture was then neutralized with aqueous NaHCO_3_ solution, the solvent was evaporated and the residue diluted with water and extracted with DCM. The combined organic extract was dried over anhydrous MgSO_4_ and evaporated. The residue was purified by VLC (vacuum liquid chromatography, petroleum ether/ethyl acetate, 7:3) to afford **7**, as a colorless oil, 8 mg (65%). [α]_D_^24^ +56 (*c* 0.26, CHCl_3_); NMR data for modified site (C14–C19): ^1^H-NMR (CDCl_3_, 500 MHz) δ 4.85 (dd, *J* = 10.8, 2.5 Hz, 1H, H-14), 5.53 (dd, *J* = 9.7, 2.5 Hz, 1H, H-15), 3.80 (dt, *J* = 9.7, 5.3 Hz, 1H, H-16), 3.40 (dd, *J* = 8.3, 5.3 Hz, 1H, H-17), 5.31 (dd, *J* = 15.5, 8.3 Hz, 1H, H-18), 5.57 (m, 1H, H-19), 3.18 (s, 3H, OMe); ^13^C-NMR (CDCl_3_, 125 MHz) δ 77.6 d (C-14), 71.4 d (C-15), 72.5 d (C-16), 82.1 d (C-17), 128.2 d (C-18), 131.9 d (C-19), 55.9 q (OMe). HR-ESIMS *m*/*z* calculated for C_36_H_50_N_2_O_11_Na (M + Na^+^) 709.3312, found 709.3322.

#### 3.2.3. Compounds **8** and **9**

A solution of salarin C (25 mg, 0.038 mmol) in CH_3_CN:H_2_O (3:1, 0.5 mL), was stirred with cerium ammonium nitrate (41 mg, 0.076 mmol) at rt for 3 h. The solvent was then concentrated in vaccum, H_2_O and DCM were added and the organic layer was dried over anhydrous MgSO_4_ and evaporated. The residue was purified by VLC (petroleum ether/ethyl acetate, 1:1) to afford **8**, as a colorless oil, 7 mg (24%) and **9**, a second colorless oil, 8 mg (35%). **8**: [α]_D_^24^ +68 (*c* 0.26, CHCl_3_); NMR data for modified site (C14–C19): ^1^H-NMR (CDCl_3_, 500 MHz) δ 4.95 (dd, *J* = 9.3, 2.4 Hz, 1H, H-14), 5.55 (dd, *J* = 9.3, 3.9 Hz, 1H, H-15), 3.77 (ddd, *J* = 9.8, 6.2, 3.9 Hz, 1H, H-16), 4.07 (t, *J* = 6.2 Hz, 1H, H-17), 5.56 (dd, *J* = 15.8, 6.2 Hz, 1H, H-18), 5.72 (m, 1H, H-19); ^13^C-NMR (CDCl_3_, 100 MHz) δ 77.2 d (C-14), 71.6 d (C-15), 72.5 d (C-16), 70.7 d (C-17), 130.4 d (C-18), 129.5 d (C-19); FABMS *m*/*z* 673.3, (C_35_H_49_N_2_O_11_, MH^+^). **9**: [α]_D_^23^ +70 (*c* 0.2, CHCl_3_); NMR data for modified site (C14–C19): ^1^H-NMR (CDCl_3_, 400 MHz) δ 4.89 (dd, *J* = 8.6, 2.2 Hz, 1H, H-14), 5.48 (dd, *J* = 8.6, 2.2 Hz, 1H, H-15), 4.45 (m, 1H, H-16), 5.67 (dd, *J* = 15.5, 6.1 Hz, 1H, H-17), 5.74 (m, 1H, H-18), 4.15 (m, 1H, H-19); ^13^C-NMR (CDCl_3_, 100 MHz) δ 76.4 d (C-14), 72.9 d (C-15), 71.2 d (C-16), 127.3 d (C-17), 136.4 d (C-18), 67.6 d (C-19); HR-ESIMS *m*/*z* calculated for C_35_H_48_N_2_O_11_Na (M + Na^+^) 695.3150, found 695.3135.

#### 3.2.4. Compound **11**

MgBr_2_ was prepared from magnesium turnings (100 mg) in dry diethyl ether (10 mL) and dibromoethane (1.5 mL). A solution of salarin A (21 mg, 0.03 mmol) in dry diethyl ether (1 mL), was added dropwise to the 0 °C mixture of the MgBr_2_. The solution was stirred for 2.5 h, then water added and the solution extracted with diethyl ether. The organic layer was washed with brine, dried over anhydrous MgSO_4_ and the solvents were evaporated. The residue was purified by VLC (petroleum ether/ethyl acetate, 1:1) to afford a bromohydrin product which was unstable; ESIMS *m*/*z* 789.2, (C_34_H_45_BrN_2_O_12_Na, M + Na^+^). After 3 h, a second VLC (PE/EA, 1:1) afforded **11**, 8 mg (40%). NMR data for modified site (C13–C19): ^1^H-NMR (CDCl_3_, 500 MHz) δ 3.03 (brt, *J* = 2.4 Hz, 1H, H-13), 4.21 (brt, *J* = 2.4 Hz, 1H, H-14), 5.34 (dd, *J* = 5.1, 2.4 Hz, 1H, H-15), 5.04 (dd, *J* = 7.0, 5.1, 1H, H-16); 4.40 (t, *J* = 6.8 Hz, 1H, H-17); 5.56 (dd, *J* = 14.9, 6.8 Hz, 1H, H-18); 5.79 (m, 1H, H-19); ^13^C-NMR (CDCl_3_, 125 MHz) δ 55.0 d (C-13), 80.0 d (C-14), 74.5 d (C-15), 75.4 d (C-16), 80.9 d (C-17), 128.9 d (C-18), 130.2 d (C-19). FABMS *m*/*z* 709.3, (C_35_H_46_N_2_O_12_Na, M + Na^+^).

#### 3.2.5. Compound **12**

A solution of salarin C (10 mg, 0.015 mmol) in EtOH (5 mL) with catalytic amounts of Pd/C (5%) was hydrogenated for 30 min at 1 atmosphere to afford **12**, as a colorless oil, 8 mg (80%). [α]_D_^23^ −22 (*c* 0.14, CHCl_3_); NMR data for modified site (C14–20, C26): ^1^H-NMR (CDCl_3_, 500 MHz) δ 2.50 (m, 2H, H-8), 2.13 (m, 1H, H-9), 1.84 (m, 2H, H-10), 3.32 (dd, *J* *=* 4.4, 2.2 Hz, H-13), 4.77 (dd, *J* = 7.5, 2.9 Hz, 1H, H-14), 5.34 (dd, *J* = 7.5, 3.2 Hz, 1H, H-15), 2.74 (dd, *J* = 14.9, 3.2 Hz, 1Ha, H-16), 2.49 (m, 1Hb, H-16), 3.71 (m, 1H, H-17), 2.39 (m, 2H, H-18), 1.73 (m, 2H, H-19), 1.64 (m, 2H, H-20); COSY correlations and *J* values supported the identification of the later protons.

^13^C-NMR (CDCl_3_, 125 MHz) δ 172.2 s (C-1), 33.1 t (C-2), 25.7 t (C-3), 31.6 t (C-4), 23.9 t (C-5), 133.2 s (C-6), 161.2 s (C-7), 35.4 t (C-8), 29.8 d (C-9), 30.6 t (C-10), 76.3 d (C-14), 75.8 d (C-15), 34.3 t (C-16), 71.1 d (C-17), 35.1 t (C-18), 22.6 t (C-19), 28.5 t (C-20), 142.3 s (C-26). FABMS *m*/*z* 687.3, (C_35_H_56_N_2_O_10_Na, M + Na^+^).

#### 3.2.6. Compound **13**

A mixture of salarin C (30 mg, 0.043 mmol), morpholine (6 mg, 0.08 mmol) and Zn(ClO_4_)_2_·6H_2_O (0.3 mg, 2 mol%) in dry DCM (2 mL) was stirred at rt under argon in the dark for 48 h. After completion of the reaction (TLC), DCM (5 mL) was added and the mixture washed with water, dried over anhydrous MgSO_4_ and evaporated. The residue was purified by VLC (petroleum ether/ethyl acetate, 3:2) to afford **13**, as a yellow oil, 28 mg (78%). [α]_D_^27^ +14 (*c* 0.13, CHCl_3_); NMR data for modified site (C14–C19, C25): ^1^H-NMR (CDCl_3_, 500 MHz) δ 4.93 (dd, *J* = 8.9, 2.5 Hz, 1H, H-14), 5.48 (m, 1H, H-15), 3.85 (t, *J* = 9.2 Hz, 2H, H-16), 2.80 (t, *J* = 9.2 Hz, 1H, H-17), 5.27 (dd, *J* = 15.4 Hz, 9.2, 2H, H-18), 5.47 (m, 2H, H-19), 2.44 (s, 3H, H-25); morpholine moiety: 2.52 (m, 4H, CH_2_N), 3.68 (m, 4H, CH_2_O); ^13^C-NMR (CDCl_3_, 125 MHz) δ 77.8 d (C-14), 73.7 d (C-15), 66.9 d (C-16), 71.5 d (C-17), 125.7 d (C-18), 132.8 d (C-19), 23.4 d (C-25); morpholine moiety: 48.6 t (CH_2_N), 66.9 t (CH_2_O). HR-ESIMS *m*/*z* calculated for C_39_H_56_N_3_O_11_ (MH^+^) 742.3915, found 742.3917.

#### 3.2.7. Compound **14**

A mixture of salarin C (50 mg, 0.043 mmol), n-butylamine (18 mg, 0.2 mmol) and Zn(ClO_4_)_2_·6H_2_O (0.6 mg, 2 mol%) in dry DCM (4 mL) was stirred at rt under argon in the dark for 48 h. After completion of the reaction (TLC), DCM was added (5 mL), and the mixture was washed with water, dried over anhydrous MgSO_4_ and evaporated. The residue was purified by VLC (petroleum ether/ethyl acetate, 1:1) to afford **14**, as a yellow oil, 7 mg (12%). [α]_D_^27^ +30 (*c* 0.17, CHCl_3_); NMR data for modified site (C14–C19, C25): ^1^H-NMR (CDCl_3_, 500 MHz) δ 4.91 (dd, *J* = 8.8, 2.0 Hz, 1H, H-14), 5.45 (dd, *J* = 7.7, 2.0 Hz, 1H, H-15), 3.60 (t, *J* = 7.7 Hz, 1H, H-16), 2.83 (t, *J* = 7.7 Hz, 1H, H-17), 5.21 (dd, *J* = 15.0, 8.8 Hz, 1H, H-18), 5.40 (dd, *J* = 15.0, 6.5 Hz, 1H, H-19), 2.42 (s, 3H, H-25); butylamine moiety: 2.61 (m, CH_2_NH), 2.34 (m, CH_2_NH), 1.41 (m, 2H, H-2″); 1.31 (m, 2H, H-3″), 0.92 (t, *J* = 7.3 Hz, CH_3_); ^13^C-NMR (CDCl_3_, 125 MHz) δ 78.4 d (C-14), 73.8 d (C-15), 71.6 d (C-16), 64.8 d (C-17), 131.0 d (C-18), 129.9 d (C-19), 23.9 d (C-25); butylamine moiety: 46.5 t (CH_2_NH), 32.3 t (CH_2_, C-2″), 20.2 t (CH_2_, C-3″), 14.8 q (CH_3_). HR-ESIMS *m*/*z* calculated for C_39_H_58_N_3_O_10_ (MH^+^) 728.4122, found 728.4130.

#### 3.2.8. Compound **15**

A mixture of salarin C (20 mg, 0.03 mmol), benzylamine (12 mg, 0.09 mmol) and Zn(ClO_4_)_2_·6H_2_O (0.3 mg, 2 mol%) in dry DCM (4 mL) was stirred at rt under argon in the dark for 72 h. After completion of the reaction (TLC), DCM was added and the mixture was washed with water, dried over anhydrous MgSO_4_ and evaporated. The residue was purified by VLC (petroleum ether/ethyl acetate, 3:7) to afford **15**, as a yellow oil, 9 mg (36%). [α]_D_^27^ 30 (*c* 0.17, CHCl_3_); NMR data for modified site (C14–C19): ^1^H-NMR (CDCl_3_, 500 MHz) δ 4.81 (dd, *J* = 8.6, 2.0 Hz, 1H, H-14), 5.47 (m, 1H, H-15), 3.71 (m, 1H, H-16), 2.93 (t, *J* = 7.6 Hz, 1H, H-17), 5.26 (dd, *J* = 16.0, 8.1 Hz, 1H, H-18), 5.50 (m, 1H, H-19); benzylamine moiety: δ 3.78 (d, *J* = 12.8 Hz, 1Ha, H-26), 3.54 (d, *J* = 12.8 Hz, 1Hb, H-26), 7.31 (m, 2H, H-28,30), 7.31 (m, 2H, H-29,31), 7.31 (m, 1H, H-30); ^13^C-NMR (CDCl_3_, 125 MHz) δ 76.7 d (C-14), 74.3 d (C-15), 72.4 d (C-16), 64.8 d (C-17), 130.7 d (C-18), 130.9 d (C-19), 156.3 s (C-23); benzylamine moiety: 50.9 t (C-26), 140.0 s (C-27), 129.2 d (C-28,32), 129.3 d (C-29,31), 127.8 d (C-30). HR-ESIMS *m*/*z* calculated for C_40_H_54_N_3_O_9_ (MH^+^) 720.3860, found 720.3865.

#### 3.2.9. Compounds **16** and **18**

A mixture of salarin C (25 mg, 0.038 mmol), NH-Boc-hexane-amine (30 mg, 0.11 mmol) and Zn(ClO_4_)_2_·6H_2_O (0.3 mg, 2 mol%) in dry DCM (2 mL) was stirred at rt under argon in the dark for 72 h. After completion of the reaction (TLC), DCM was added and the mixture was washed with water, dried over anhydrous MgSO_4_ and evaporated. The residue was purified by VLC (petroleum ether/ethyl acetate, 3:7) to afford **16**, as a colorless oil, 8 mg (25%) and **18**, a second colorless oil, 4 mg (13%). **16**: [α]_D_^27^ +50 (*c* 0.15, CHCl_3_); NMR data for modified site (C14–C19, C23): ^1^H-NMR (CDCl_3_, 500 MHz) δ 4.85 (d, *J* = 9.0 Hz, 1H, H-14), 5.43 (m, 1H, H-15), 3.61 (brt, *J* = 7.0 Hz, 1H, H-16), 2.86 (brt, *J* = 7.0 Hz, 1H, H-17), 5.27 (m, 1H, H-18), 5.541 (m, 1H, H-19); NH-Boc-diaminehexane moiety: 2.62 (m, 2H, CH_2_NH), 2.35 (m, 2H, CH_2_NH), 1.40 (m, 2H), 1.32 (m, 2H), 1.30 (m, 2H), 1.40 (m, 2H), 3.10 (m, 2H, CH_2_NH-Boc), 1.44 (s, 9H, CH_3_); ^13^C-NMR (CDCl_3_, 125 MHz) δ 75.1 d (C-14), 72.5 d (C-15), 70.4 d (C-16), 63.2 d (C-17), 130.6 d (C-18), 129.4 d (C-19); 155.1 s (C-23); NH-Boc-diaminehexane moiety: δ 45.4 t (CH_2_NH), 29.6 t (CH_2_), 25.5 t (CH_2_), 25.4 t (CH_2_), 29.6 t (CH_2_), 39.2 t (CH_2_NH-Boc), 155.6 s (CO), 78.9 s (C), δ_C_ 26.7 q (3CH_3_). HR-ESIMS *m*/*z* calculated for C_44_H_70_N_4_O_11_ (MH^+^) 829.4963, found 829.4969; **18**: [α]_D_^27^ +53 (*c* 0.13, CHCl_3_); NMR data for modified site (C14–C19, C23): ^1^H-NMR (CDCl_3_, 500 MHz) δ 3.94 (m, 1H, H-14), 5.27 (m, 1H, H-15), 3.69 (brt, *J* = 7.1 Hz, 1H, H-16), 2.92 (brt, *J* = 7.5 Hz, 1H, H-17), 5.31 (dd, *J* = 15.1, 7.5 Hz, 1H, H-18), 5.45 (dt, *J* = 15.1, 6.3 Hz, 1H, H-19); NH-Boc-diaminehexane moiety: 2.61 (m, 1Ha, CH_2_NH), 2.34 (m, 1Hb, CH_2_NH), 1.48 (m, 2H), 1.34 (m, 2H), 1.30 (m, 2H), 1.40 (m, 2H), 3.11 (m, 2H, CH_2_NH-Boc), 1.48 (s, 9H, CH_3_); ^13^C-NMR (CDCl_3_, 125 MHz) δ 71.5 d (C-14), 75.8 d (C-15), 71.8 d (C-16), 63.3 d (C-17), 125.5 d (C-18), 130.6 d (C-19); NH-Boc-diaminehexane moiety: 45.9 t (CH_2_NH), 30.0 t (CH_2_), 25.5 t (CH_2_), 25.5 t (CH_2_), 30.3 t (CH_2_), 39.8 t (CH_2_NH-Boc), 156.0 s (CO), 79.0 s (C), δ_C_ 27.0 q (3CH_3_). HR-ESIMS *m*/*z* calculated for C_43_H_68_N_3_O_10_ (MH^+^) 786.4905, found 786.4918.

#### 3.2.10. Compound **17**

A mixture of salarin C (16 mg, 0.024 mmol), 1,6-diaminohexane (8.5 mg, 0.07 mmol) and Zn(ClO_4_)_2_·6H_2_O (0.2 mg, 2 mol%) at 60 °C, neat, was stirred under argon in the dark for 20 min. After completion of the reaction (TLC), DCM was added and the mixture was washed with water, dried over anhydrous MgSO_4_, and evaporated to afford **17**, as a colorless oil, 13 mg (70%). [α]_D_^27^ +50 (*c* 0.3, CHCl_3_); NMR data for modified site (C14–C19, C25): ^1^H-NMR (CDCl_3_, 500 MHz) δ 4.84 (d, *J* = 9.3 Hz, 1H, H-14), 5.47 (d, *J* = 9.3 Hz, 1H, H-15), 3.60 (brt, *J* = 7.6 Hz, 1H, H-16), 2.82 (brt, *J* = 7.6 Hz, 1H, H-17), 5.23 (dd, *J* = 15.3, 9.1 Hz, 1H, H-18), 5.42 (m, 1H, H-19), 2.01 (s, 3H, H-25); 1,6-diaminehexane moiety: δ 2.62 (m, 1Ha, CH_2_NH), 2.31 (m, 1Hb, CH_2_NH), 1.44 (m, 2H), 1.32 (m, 2H), 1.30 (m, 2H), 1.44 (m, 2H), 2.69 (m, 2H, CH_2_NH_2_); ^13^C-NMR (CDCl_3_, 125 MHz) δ 75.4 d (C-14), 73.1 d (C-15), 70.1 d (C-16), 63.1 d (C-17), 130.8 d (C-18), 129.0 d (C-19), 155.7 s (C-23), 170.2 s (C-24), 24.8 q (C-25); 1,6-diaminehexane moiety: δ 45.8 t (CH_2_NH), 32.5 t (CH_2_), 26.1 t (CH_2_), 26.1 (CH_2_), 32.5 t (CH_2_), 41.3 t (CH_2_NH_2_); HR-ESIMS *m*/*z* calculated for C_41_H_63_N_4_O_10_ (MH^+^) 771.4544, found 771.4547.

#### 3.2.11. Compound **19**

A mixture of salarin A (30 mg, 0.043 mmol), morpholine (6 mg, 0.08 mmol) and Zn(ClO_4_)_2_·6H_2_O (0.3 mg, 2 mol%) in dry DCM (2 mL) was stirred at rt under argon. After completion of the reaction (72 h, TLC), the mixture was diluted with DCM (5 mL), washed with water, dried over anhydrous MgSO_4_ and then evaporated. The crude residue was purified by VLC (petroleum ether/ethyl acetate, 4:6) to afford **19**, as a yellow oil, 28 mg (78%). [α]_D_^23^ −30 (*c* 0.16, CHCl_3_); NMR data for modified site (C1–7, C26,27): ^1^H-NMR (CDCl_3_, 500 MHz) δ 5.90 (m, 1H, H-2), 6.70 (t, *J* = 11.8 Hz, 1H, H-3), 8.19 (dd, *J* = 15.0, 11.8 Hz, 1H, H-4), 6.57 (d, *J* = 15.0 Hz, 1H, H-5), 2.34 (s, 3H, H-27), 8.15 (s, 1H, NH); Morpholine: 3.71 (m, 4H, 2 × CH_2_O), 3.74 (m, 4H, 2 × CH_2_NH); ^13^C-NMR (CDCl_3_, 100 MHz) δ 164.1 s (C-1), 121.9 d (C-2), 140.0 d (C-3), 137.4 d (C-4), 126.6 d (C-5), 164.0 s (C-6), 163.6 s (C-7), 171.9 s (C-26), 23.8 q (C-27); Morpholine: 66.0 t (CH_2_O), 42.5 t (CH_2_NH); FABMS *m*/*z* 796.1, (C_39_H_55_N_3_O_13_Na, M + Na^+^).

#### 3.2.12. Compound **20**

To a solution of salarin C (10 mg, 0.015 mmol) in MeOH (2 mL) was added aqueous ammonia (0.5 mL). The mixture was stirred 3 h at rt and then evaporated. The residue was purified by VLC (petroleum ether/ethyl acetate, 1:1) to afford **20**, as a colorless oil, 7 mg (87%). [α]_D_^25^ −38 (*c* 0.17, CHCl_3_); NMR data for modified site (C13–C15, C23): ^1^H-NMR (CDCl_3_, 500 MHz) δ 3.43 (dd, *J* = 8.6, 1.8 Hz, 1H, H-13), 4.60 (m, 1H, H-14), 5.44 (dd, *J* = 8.6, 2.6 Hz, 1H, H-15); ^13^C-NMR (CDCl_3_, 125 MHz) δ 55.1 d (C-13), 76.4 d (C-14), 68.3 d (C-15), 155.2 s (C-23). HR-ESIMS *m*/*z* calculated for C_33_H_44_N_2_O_9_Na (M + Na^+^) 635.2945, found 635.2947.

#### 3.2.13. Compound **21**

To a solution of salarin C (20 mg, 0.03 mmol) in MeOH (0.5 mL) was added methanolic K_2_CO_3_ (5% v/v, 1 mL). The reaction was stirred for 2 h at rt and then EA (5 mL) was added, the organic layer was washed with saturated NH_4_Cl, dried over anhydrous Na_2_SO_4_ and evaporated. The residue was purified by VLC (petroleum ether/ethyl acetate, 3:7) to afford **21**, as a major product, colorless oil, 9 mg (62%) accompanied with traces of **20** and (2*Z*,4*E*)-dimethyl hexa-2,4-dienedioate; NMR data for modified site (C18–C21, C23): ^1^H-NMR (CDCl_3_, 400 MHz) δ 5.54 (dd, *J* = 4.1, 15.6 Hz, 1H, H-18), 5.86 (ddd, *J* = 15.6, 8.8, 5.2 Hz, 1H, H-19), 2.33 (m, 2H, H-20), 3.67 (m, 1Ha, H-21), 3.59 (ddd, *J* = 11.7, 6.8, 4.1 Hz, 1Hb, H-21); ^13^C-NMR (CDCl_3_, 100 MHz) δ 123.9 d (C-18), 133.7 d (C-19), 36.2 t (C-20), 61.5 t (C-21), 159.1 s (C-23). HR-ESIMS *m*/*z* calculated for C_25_H_30_N_2_O_8_Na (M + Na^+^) 509.1900, found 509.1898.

#### 3.2.14. Compound **22**

To a solution of salarin A (11 mg, 0.016 mmol) in EtOH (5 mL) was added NH_4_Cl (2.5 mg, 0.048 mmol) and NaN_3_ (3.1 mg, 0.048 mmol). The solution was slowly warmed up to 60 °C for 1 h, and then allowed to cool down to room temperature. The reaction mixture was filtered off, the solid residue washed with EtOH, and the solvent was evaporated. The residue was dissolved in DCM (50 mL) and washed with H_2_O (30 mL). The organic layer was dried over anhydrous Na_2_SO_4_ and then evaporated to afford **22**, as a yellow oil, 8 mg (80%). The NMR-data is identical to natural salarin E [6].

#### 3.2.15. Compound **23**

To a solution of salarin A (10 mg, 0.015 mmol) in MeOH (2 mL), was added NH_4_OH (0.5 mL, 0.1 mmol). The reaction mixture was stirred for 3 h at room temperature and then evaporated. The residue was purified by LH-20 chromatography (petroleum ether/DCM/MeOH, 2:1:1) to afford **23**, as a yellow oil, 7.5 mg (78%). [α]_D_^23^ −21 (*c* 0.2, CHCl_3_); NMR data for modified site (C1–9, C14, C22,23): ^1^H-NMR (CDCl_3_, 500 MHz) δ 5.98 (d, *J* = 11.2 Hz, 1H, H-2), 6.68 (t, *J* = 11.2 Hz, 1H, H-3), 8.29 (dd, *J* = 15.3, 11.2 Hz, 1H, H-4), 6.13 (d, *J* = 15.3 Hz, 1H, H-5), 5.66 (d, *J* = 8.4 Hz, 1H, H-8), 4.90 (dd, *J* = 6.6, 3.6 Hz, 1H, H-14), 1.88 (s, 3H, H-22), 3.78 (s, 3H, OMe); δ ^13^C-NMR (CDCl_3_, 125 MHz) δ 163.9 s (C-1), 124.1 d (C-2), 141.5 d (C-3), 138.9 d (C-4), 129.3 d (C-5), 166.8 s (C-6), 168.7 s (C-7), 118.7 d (C-8), 155.7 s (C-9), 74.3 d (C-14), 25.1 q (C-22), 155.8 s (C-23), 52.3 q (OMe). HR-ESIMS *m*/*z* calculated for C_32_H_46_N_2_O_11_Na (M + Na^+^) 657.2999, found 657.3004.

#### 3.2.16. Compound **24**

To a solution of **21** (20 mg, 0.04 mmol) in DCM (2 mL) was added triethylamine (20 μL, 0.12 mmol), DMAP (0.48 mg, 0.004 mmol) and bis(4-nitrophenyl) carbonate (20 mg, 0.06 mmol). The mixture was stirred for 2 h at rt and then DCM was added and the mixture was washed with brine. The organic layer was dried over anhydrous MgSO_4_ and evaporated. The residue was purified by VLC (petroleum ether/ethyl acetate, 4:6) to afford **24**, as a colorless oil, 12 mg (46%). [α]_D_^25^ +117 (*c* 0.07, CHCl_3_); NMR data for modified site (C21-PNP): ^1^H-NMR (CDCl_3_, 400 MHz) δ 4.38 (t, *J* = 6.6 Hz, 2H, H-21); PNP moiety: δ 8.27 (m, *J* = 8.8 Hz, 1H, H-2′), 7.34 (m, *J* = 8.8 Hz, 1H, H-3′); ^13^C-NMR (CDCl_3_, 100 MHz) δ 68.2 t (C-21); PNP moiety: δ 152.15 s (CO), 155.3 s (C-1′), 121.9 d (C-2′), 125.2 d (C-3′), 145.9 s (C-4′); ESIMS *m*/*z* 674.45, (C_32_H_33_N_3_O_12_Na, M + Na^+^).

#### 3.2.17. Compound **25**

To a mixture of d(+)-glucosamine and NaOMe (7 mg, 0.035 mmol) in dry DMF (0.5 mL) was added Et_3_N (15 μL, 0.1 mmol) followed by **24** (10 mg, 0.014 mmol) in dry DMF (0.2 mL) and the mixture was stirred for 2 h at rt. The solvent was evaporated and the residue was purified by LH-20 chromatography (petroleum ether/DCM/MeOH, 2:1:1) to afford **25**, as a colorless oil, 8 mg (80%). [α]_D_^25^ +206 (*c* 0.2, MeOH); NMR data for modified site (C14–C19): ^1^H-NMR (*d*_4_-MeOH, 500 MHz) δ 5.13 (d, *J* = 3.8 Hz, 1H, H-1′), 4.12 (m, 2H, H-21), 3.57 (m, *J* = 10.4, 3.8 Hz, H-2′), 3.64 (m, 1H, H-3′), 3.89 (m, 1H, H-4′), 3.39 (m, 1H, H-5′), 3.63 (m, 1Ha, H-6′), 3.69 (m, 1Hb, H-6′); ^13^C-NMR (*d*_4_-MeOH, 125 MHz) δ 63.2 t (C-21), 155.9 s (CO), 91.3 d (C-1′), 56.5 d (C-2′), 71.6 d (C-3′), 71.4 d (C-4′), 70.8 d (C-5′), 61.4 t (C-6′). HR-ESIMS *m*/*z* calculated for C_32_H_41_N_3_O_14_Na (M + Na^+^) 714.2486, found 714.2489.

### 3.3. Tulearin Derivatives

#### 3.3.1. Compounds **26** and **27**

To a mixture of tulearin A (222 mg, 0.415 mmol) and pyridine (1 mL) in chloroform (3 mL), was added TsCl (160 mg, 0.8 mmol) at 0 °C. After 48 h of stirring, EA was added and the organic phase was washed with saturated NH_4_Cl, water and brine, dried over anhydrous MgSO_4_ and evaporated. The residue was purified by VLC (petroleum ether/ethyl acetate, 1:1) to afford ditosyl **26**, as a colorless oil, 52 mg (20%) and 9-monotosyl **27**, a second colorless oil, 43 mg (35%). **26**: [α]_D_^25^ +15 (*c* 0.4, CHCl_3_); NMR data for modified site (C3–C10): ^1^H-NMR (CDCl_3_, 500 MHz) δ 4.50 (m, 1H, H-3), 1.56 (m, 1H, H-4), 4.78 (m, 1H, H-8), 4.69 (m, 1H, H-9), 1.57 (m, 1H, H-10);. 3-Ts: δ 7.35 (d, 2H, H-33,34), 7.79 (d, 2H, H-35,36), 2.45 (s, 3H, H-38); 9-Ts: δ 7.35 (d, 2H, H-33,34), 7.79 (d, 2H, H-35,36), 2.45 (s, 3H, H-38); ^13^C-NMR (CDCl_3_, 125 MHz) δ 80.4 d (C-3), 35.3 t (C-4), 72.1 d (C-8), 81.5 d (C-9), 31.3 t (C-10); 3-Ts: δ 127.5 s (C-32), 129.9 d (C-33,34), 127.8 d (C-35,36), 137.0 s (C-37), 21.0 q (C-38); 9-Ts: δ 127.5 s (C-32), 129.9 d (C-33,34), 127.8 d (C-35,36), 137.0 s (C-37), 21.0 q (C-38). HR-ESIMS *m*/*z* calculated for C_45_H_65_NO_10_S_2_Na (M + Na^+^) 866.3948, found 866.3954. **27**: [α]_D_^25^ +17 (*c* 0.2, CHCl_3_); NMR data for modified site (C8–C10): ^1^H-NMR (CDCl_3_, 500 MHz) δ 4.78 (m, 1H, H-8), 4.69 (m, 1H, H-9), 1.57 (m, 1H, H-10); 9-monoTs: δ 7.35 (d, 2H, H-33,34), 7.79 (d, 2H, H-35,36), 2.45 (s, 3H, H-38); ^13^C-NMR (CDCl_3_, 125 MHz) δ 72.1 d (C-8), 81.5 d (C-9), 31.3 t (C-10); 9-monoTs: δ 127.5 s (C-32), 129.9 d (C-33,34), 127.8 d (C-35,36), 137.0 s (C-37), 21.0 q (C-38); HR-ESIMS *m*/*z* calculated for C_38_H_59_NO_8_NaS (M + Na^+^) 712.3859, found 712.3860.

#### 3.3.2. Compound **28**

To a solution of tulearin A (10 mg, 0.025 mmol) in DCM (2 mL) were added Et_3_N (5.2 mg, 0.05 mmol) and *p*-bromobenzoylchloride (11.3 mg, 0.05 mmol). The mixture was stirred at room temperature for 24 h. DCM was then added (5 mL) and the mixture was washed with saturated NH_4_Cl, water and brine, dried over anhydrous MgSO_4_ and evaporated. The residue was purified by VLC (petroleum ether/ethyl acetate, 1:1) to afford **28**, as a colorless oil, 7.2 mg (40%). [α]_D_^25^ +27 (*c* 0.2, CHCl_3_); NMR data for modified site (C8–C10): ^1^H-NMR (CDCl_3_, 500 MHz) δ 4.95 (q, *J* = 5.3 Hz, 1H, H-8), 5.29 (q, *J* = 5.3 Hz, 1H, H-9), 1.62 (m, 2H, H-10); Aryl group (Ar): δ 7.90 (d, *J* = 8.3 Hz, 2H, H-34,35), 7.57 (d, *J* = 8.3 Hz, H-36,37); ^13^C-NMR (CDCl_3_, 125 MHz) δ 74.3 d (C-8), 73.4 d (C-9), 31.4 t (C-10); Aryl group (Ar): δ 165.2 s (C-32), 137.0 s (C-33), 131.9 d (C-34,35), 131.4 d (C-36,37), 128.0 s (C-38). FABMS *m*/*z* 718.0, (C_38_H_57_BrNO_7_, MH^+^).

#### 3.3.3. Compound **29**

Mesyl chloride (80 mg, 0.7 mmol) was added to a cold solution (0 °C) of tulearin A (140 mg, 0.26 mmol) in pyridine (0.14 mL). The mixture was stirred at rt for 3 h. Then, water was added and the mixture extracted with DCM. The organic phase was dried over anhydrous MgSO_4_ and evaporated. The residue was purified by VLC (petroleum ether/ethyl acetate, 4:1) to afford **29**, as a colorless oil, 110 mg (61%). [α]_D_^25^ +15 (*c* 0.5, CHCl_3_); NMR data for modified site: ^1^H-NMR (CDCl_3_, 500 MHz) δ 5.00 (brd, *J* = 9.0 Hz, 1H, H-3), 3.08 (s, CH_3_-Ms), 5.21 (brd, *J* = 8.9 Hz, 1H, H-9), 3.05 (s, 3H, CH_3_-Ms); ^13^C-NMR (CDCl_3_, 125 MHz) δ 80.6 d (C-3), 40.9 q (CH_3_-Ms), 80.9 d (C-9), 38.9 q (CH_3_-Ms); CIMS *m*/*z* 692.0, (C_33_H_58_NO_10_S_2_, MH^+^).

#### 3.3.4. Compound **30**

To a solution of tulearin A (22 mg, 0.044 mmol) in DCM (0.5 mL) were added Et_3_N (18 μL, 0.13 mmol) and catalytic amounts of DMAP at 0 °C. Benzoyl chloride was then added (20 μL, 0.17 mmol) and the mixture was warmed up slowly to rt. After 2.5 h, the reaction mixture was neutralized by acetic acid to pH 7 and partitioned between ethyl acetate/H_2_O. The organic layer was washed with sat. NH_4_Cl, dried over anhydrous MgSO_4_ and evaporated. The residue was purified by VLC (PE/EA, 1:1) to afford **30**, as a colorless oil, 19 mg (70%). NMR data for modified site (C8–C10): ^1^H-NMR (CDCl_3_, 500 MHz) δ 5.02 (dt, *J* = 5.6, 6.2 Hz, 1H, 1H, H-8), 5.37 (dt, *J* = 5.6, 5.9 Hz, 1H, H-9), 1.78 (m, 2H, H-10); Benzoyl group: δ 7.57 (d, *J* = 7.8 Hz, 1H), 8.06 (d, *J* = 7.8 Hz, 2H), 7.45 (t, *J* = 7.8 Hz, 2H); ^13^C-NMR (CDCl_3_, 125 MHz) δ 73.9 d (C-8), 72.55 d (C-9), 28.9 t (C-10); Benzoyl group: δ 166.6 s (CO), 132.6 d (CH), 129.4 d (CH, 2H), 127.9 d (CH, 2H); HR-ESIMS *m*/*z* calculated for C_38_H_57_NO_7_Na (M + Na^+^) 662.4033, found 662.4030.

#### 3.3.5. Compound **31**

To a mixture of pyridine (0.1 mL, 1.06 mmol) and acetic anhydride (0.1 mL, 1.2 mmol) was added tulearin A (43 mg, 0.086 mmol) at rt. After 20 min the solvents were evaporated and the residue was purified by VLC (petroleum ether/ethyl acetate, 3:2) to afford **31**, as major product, accompanied by 10% of **32**, and 10% tulearin A. **31**: colorless oil, 30 mg (61%); [α]_D_^25^ +27 (*c* 0.3, CHCl_3_); NMR data for modified site (C7–C10): ^1^H-NMR (CDCl_3_, 500 MHz) δ 1.62 (m, 2H, H-7), 4.84 (dt, *J* = 10.5, 5.8 Hz, 1H, H-8), 5.13 (dd, *J* = 6.5, 10.5 Hz, 1H, H-9), 1.47 (m, 2H, H-10); acetate group: δ 2.09 (s, CH3); ^13^C-NMR (CDCl_3_, 125 MHz) δ 29.5 t (C-7), 73.9 d (C-8), 71.2 d (C-9), 29.7 t (C-10); acetate group: δ 171.0 s (CO), 19.9 q (CH_3_); HR-ESIMS *m*/*z* calculated for C_33_H_55_NO_7_Na (M + Na^+^) 600.3876, found 600.3876.

#### 3.3.6. Compound **32**

To a mixture of pyridine (0.5 mL, 5.3 mmol) and acetic anhydride (0.5 mL, 6.0 mmol), was added tulearin A (20 mg, 0.04 mmol) at rt. After 20 min the solvents were evaporated and the residue was purified by VLC (petroleum ether/ethyl acetate, 4:1) to afford **32**, as a colorless oil, 22 mg, (90%); [α]_D_^25^ +26 (*c* 0.3, CHCl_3_); NMR data for modified site C3 and C9: ^1^H-NMR (CDCl_3_, 500 MHz) δ 5.19 (m, 1H, H-3); 3-acetate group: δ 2.10 (s, 3H, CH_3_), 5.10 (q, *J* = 6.7 Hz, 1H, H-9); 9-acetate group: δ 2.11 (s, 3H, CH_3_); ^13^C-NMR (CDCl_3_, 125 MHz) δ 71.6 d (C-3); 3-acetate group: δ 170.5 s (CO), 21.3 q (CH_3_); 71.3 d (C-9); 9-acetate group: δ 170.1 s (CO), 21.1 q (CH_3_); FABMS *m*/*z* 620.9, (C_35_H_58_NO_8_, MH^+^).

#### 3.3.7. Compound **33**

To a mixture of pyridine (0.1 mL, 1.06 mmol) and acetic anhydride (0.1 mL, 1.02 mmol) with catalytic amounts of DMAP (0.43 mg, 0.004 mmol), was added tulearin A (18 mg, 0.036 mmol) at rt. After 7 days the solvents were evaporated and the residue was purified by VLC (petroleum ether/ethyl acetate, 4:1) to afford **33**, as major product, accompanied by 10% of **32**; **33**: colorless oil, 9 mg (40%). NMR data for modified site: ^1^H-NMR (CDCl_3_, 500 MHz) δ 5.18 (m, 1H, H-3), 5.1 (m, 1H, H-9); 9-acetate group: δ 2.03 (s, 3H, CH_3_); 3-acetate group: δ 2.01 (s, 3H, CH_3_); acetyl carbamate: δ 2.36 (s, 3H, CH_3_); ^13^C-NMR (CDCl_3_, 125 MHz) δ 71.4 d (C-3), 76.1 d (C-9); 9-acetate group: δ 172.1 s (CO), 21.0 q (CH_3_); 3-acetate group: δ 171.6 s (CO), 20.9 q (CH_3_); acetyl carbamate: δ 170.8 s (CO), 22.5 q (CH_3_); HR-ESIMS *m*/*z* calculated for C_37_H_59_NO_9_Na (M + Na^+^) 684.4088, found 684.4084.

#### 3.3.8. Compound **34**

To a mixture of Et_3_N (13 μL, 0.096 mmol) with catalytic amounts of DMAP (0.3 mg, 0.003 mmol) in DCM (0.2 mL), was added tulearin A (12 mg, 0.024 mmol) at 0 °C. Hexanoyl chloride was then slowly added (13 μL, 0.096 mmol) and the mixture warmed up slowly to rt. After 2.5 h, the reaction mixture was neutralized by acetic acid to pH 7 and the residue was partitioned between ethyl acetate/H_2_O. The organic layer washed by sat. NH_4_Cl, dried over anhydrous MgSO_4_ and evaporated. The residue was purified by VLC (petroleum ether/ethyl acetate, 4:1) to afford **34**, as a colorless oil, 6 mg (35%). NMR data for modified site: ^1^H-NMR (CDCl_3_, 500 MHz) δ 5.15 (dt, *J* = 5.8, 6.7 Hz, 1H, H-9), 5.26 (m, 1H, H-3); 14 methylenes according to HSQC experiments in the range of 1–2.5 ppm; ^13^C-NMR (CDCl_3_, 125 MHz) δ 71.0 d (C-9), 71.2 d (C-3); Hexanoyl carbonyls: 172.0 s (C3-CO), 172.3 s (C9-CO), 173.3 s (carbamate), HR-ESIMS *m*/*z* calculated for C_49_H_83_NO_9_Na (M + Na^+^) 852.5966, found 852.5969.

#### 3.3.9. Compound **35**

To a solution of tulearin A (35 mg, 0.065 mmol) in dry DCM (3 mL) were added of *p*-nitrophenyl chlorocarbonate (PNPCl, 15 mg, 0.074 mmol) and DMAP (18 mg, 0.14 mmol). The reaction was stirred at rt for 48 h. DCM was then added (5 mL) and the mixture was washed with water, dried over anhydrous MgSO_4_ and evaporated. The residue was purified by VLC (petroleum ether/ethyl acetate, 1:1) to afford **35**, as a colorless oil, 15 mg (35%). [α]_D_^25^ +10 (*c* 0.3, CHCl_3_); NMR data for modified site (C2, C3): ^1^H-NMR (CDCl_3_, 500 MHz) δ 2.70 (qd, *J* = 7.6, 2.4 Hz, 1H, H-2), 5.09 (dd, *J* = 10.1, 2.4 Hz, 1H, H-3); PNP-group: δ 6.48 (d, *J* = 7.6 Hz, 2H, CH), 8.23 (d, *J* = 7.6 Hz, 2H, CH); ^13^C-NMR (CDCl_3_, 100 MHz) δ 44.5 d (C-2), 72.6 d (C-3); PNP-group: δ 156.6 s (CO), 130.2 q (C), 106.6 d (CH), 149.6 d (CH), 136.2 s (C). ESIMS *m*/*z* 723.3, (C_38_H_56_N_2_O_10_Na, M + Na^+^).

#### 3.3.10. Compounds **36** and **37**

To a solution of **29** (22 mg, 0.03 mmol) in DMF (0.5 mL) was added sodium azide (10 mg, 0.15 mmol), and the mixture warmed up to 60 °C for. After 24 h, the reaction mixture was cooled down to room temperature. The mixture was then partitioned between ethyl acetate/H_2_O and the organic phase dried over anhydrous MgSO_4_, and evaporated. The DMF was removed under high vacuum and the residue was purified by VLC (petroleum ether/ethyl acetate) to afford three products of azide substituted tulearin A; two epmeric 3,9-diazide tulearins: **36a**, as a colorless oil, 3.8 mg (21%); **36b**, a second colorless oil, 5.1 mg (29%) and a cycloaddition product **37**, another colorless oil, 6.3 mg (37%); **36a**: [α]_D_^25^ +20 (*c* 0.2, CHCl_3_); NMR data for modified site: ^1^H-NMR (CDCl_3_, 500 MHz) δ 3.59 (ddd, *J* = 8.7, 6.8, 2.0 Hz, 1H, H-3), 3.29 (td, *J* = 7.5, 3.7 Hz, 1H, H-9); ^13^C-NMR (CDCl_3_, 125 MHz) δ 62.3 d (C-3), 62.7 d (C-9); HR-ESIMS *m*/*z* calculated for C_31_H_51_N_7_O_4_Na (M + Na^+^) 608.3900, found 608.3898; **36b**: [α]_D_^25^ −26 (*c* 0.24, CHCl_3_); NMR data for modified site: ^1^H-NMR (CDCl_3_, 500 MHz) δ 3.63 (ddd, *J* = 8.7, 6.6, 1.5 Hz, 1H, H-3), 3.47 (dt, *J* = 9.5, 3.5 Hz, 1H, H-9); ^13^C-NMR (CDCl_3_, 125 MHz) δ 62.8 d (C-3), 60.1 d (C-9); HR-ESIMS *m*/*z* calculated for C_31_H_51_N_7_O_4_Na (M + Na^+^) 608.3900, found 608.3904; **37**: [α]_D_^25^ +69 (*c* 0.7, CHCl_3_); NMR data for modified site: ^1^H-NMR (CDCl_3_, 500 MHz) δ 3.77 (ddd, *J* = 6.7, 4.6, 2.0 Hz, 1H, H-3), 1.88 (m, 2H, H-4), 5.02 (ddd, *J* = 7.4, 4.3, 2.7 Hz, 1H, H-8), 4.24 (m, 1H, H-9), 1.92 (m, 1Ha, H-10), 1.81 (m, 1Hb, H-10), 2.36 (m, 2H, H-11), 2.32 (m, 2H, H-13); ^13^C-NMR (CDCl_3_, 125 MHz) δ 61.8 d (C-3), 39.8 t (C-4), 76.0 d (C-8), 74.9 d (C-9), 24.2 t (C-10), 38.3 t (C-11), 179.2 s (C-12), 30.1 t (C-13); HR-ESIMS *m*/*z* calculated for C_31_H_52_N_5_O_4_ (MH^+^) 558.4019, found 558.4011.

## 4. Conclusions

*Cell Viability*

The availability of the ten natural salarins (A–J) and the above synthesized derivatives, which differ in particular chemical moieties, enabled a preliminary SAR study (see Supporting Information). In respect to this, the finding that salarin C is more potent than salarin A, which differs only in the oxazole ring, suggests that this heterocycle is essential for activity. However, salarins F and I, which contain the oxazole ring but differ in the macrolide functional groups, do not display inhibitory activity on cell viability and proliferation [4]. Hence, biological activity of salarin C may rely on other moieties in addition to the heterocycle, or a combination of several moieties. In this article we report a preliminary biological evaluation of the above derivatives against human leukemic cell line K562.

It was found that the 16,17-vinyl epoxide of salarin C contributes significantly to the K562 inhibition (e.g., **5**–**13**). However, this conclusion is not straightforward, namely, when the 16,17-epoxide was replaced by a 16-hydroxy-17-butylamine (**14**), the inhibition of K562 cell viability rose to 90% (based on different cell behavior). It is suggested that the butylamine group contributes to the activity of salarin C in a different, not yet identified, mechanism. Furthermore, compound **16** is also relatively active and the most active of the hydroxyl amine derivatives **16**–**18** (Scheme 4). Again, this may be the result of another mechanism. The *N*-desacetylation (e.g., **16**) affects, but does not abrogate the activity and therefore was not found as crucial for it (**15**).

Alcohol **21** showed excellent results with similar activity to the natural product (1 μM inhibited viability of the K562 leukemia cells by more than 90%, see Supporting Information), *i.e.*, the octanoate ester is not crucial for the activity. The more polar glycosylation products, compounds **24** and **25**, showed similar activity to **21**. This section demonstrates that structural changes in the natural product are moderately effective in improving the efficacy of the molecule in inhibiting the viability of leukemia cells.

As for the tulearin derivatives, no significant changes were found for most of the compounds. The only exceptions to this were compounds **28** (showing 70% inhibition for K562 cells in comparison to tulearin **A** with 55%–60% inhibition) and **35** (92% inhibition), the former carrying a leaving group at position 9 and the latter at position 3. It is difficult to know whether the change in activity results from the change in the functional groups or from the solubility.

*Inter alia*, the mono-substituted derivatives were found to be more active than the di-substituted ones. For example, mono-acetate **31** was found to inhibit K562 cells, more than the di-acetate **32** (inhibition of 28% compared to 6%) (Table 1). Unexpectedly, the tri-acetate **33** was even more active (45% inhibition). The acetyl carbamate functionality was found to slightly increase the cytotoxicity and to inhibit K562 better than the mono- and di-acetate. In comparison to the tri-substituted acetate, the tri-hexanoyl **34** showed weaker inhibition (9% inhibition).

## Supplementary Files

Supplementary File 1 Supporting Information (PDF, 978 KB)
