# Supplementary material for: Derivatives of Salarin A, Salarin C and Tulearin A—Fascaplysinopsis sp. Metabolites
Source: Mar Drugs. 2013 Nov 11;11(11):4487–509. doi: 10.3390/md11114487 (PMC3853741; doi:10.3390/md11114487)

Supporting Information

Experimental Section

General experimental procedures. Optical rotations were obtained with a Jasco P-1010 polarimeter. IR spectra were obtained with a Bruker FTIR Vector 22 spectrometer.

^{1}H and ^{13}C NMR spectra were recorded on Bruker Avance-400 and 500 spectrometers. COSY, HMQC, NOESY, ROESY and HMBC were recorded using standard Bruker pulse sequences. FABMS measurements were recorded on a Fisons, Autospec Q instrument. Electrospray MS measurments were performed on an Applied Biosystem Q-STAR Pulsar instrument (ESI-QqTOF).

Colorimetric MTT assay. Cells (4×10^{3}) were seeded in triplicate into 96-well, flat-bottom culture plates and grown in the presence of salarin C at different concentrations for 24, 48 (data not shown) and 72 h. Untreated cells served as control. After incubation with the compound, cell growth was determined using the colorimetric methylthiazole tetrazolium bromide (MTT) assay [4]. Briefly, MTT was added to a final concentration of 5 μg/mL to each well and further incubated for 4 h at 37 ℃. After complete solubilization of the dye by acid/alcohol (0.04 N HCl in 2-propanol), plates were read at 570 nm in an ELISA reader, reference 690 nm. Growth of cells exposed to treatment was calculated as the percent of optical density (OD) of compound-treated cells to that of control cells. Graphs represent the mean results ±SEM of three identical experiments.

Figure S1. Cytotoxcity of salarin C derivatives (1 μM, 72 h).

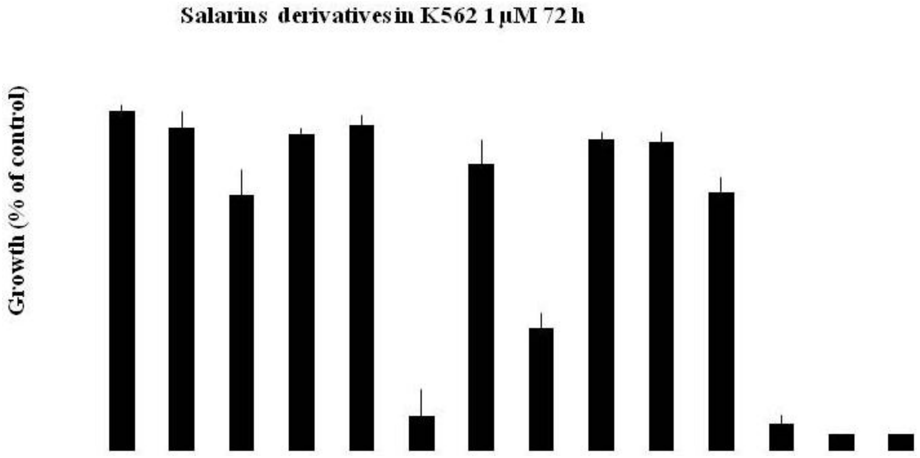

Figure S2. Cytotoxcity of several tulearin derivatives (1 μM, 72 h).

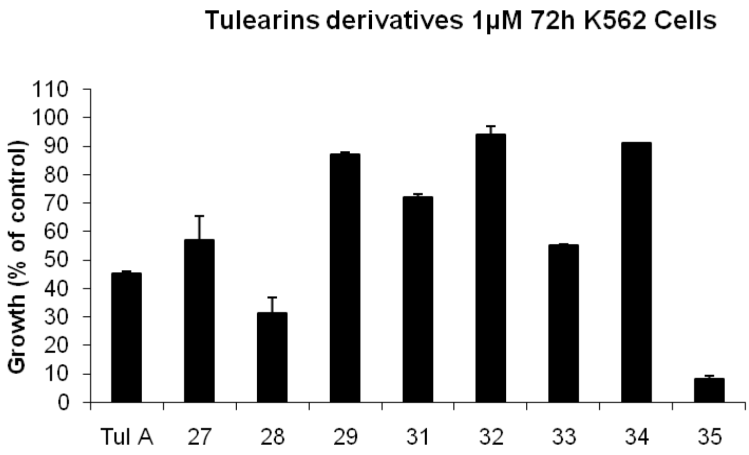

Figure S3. ^{1}H-NMR of compound **6** ($CDCl_3$, 400 MHz).

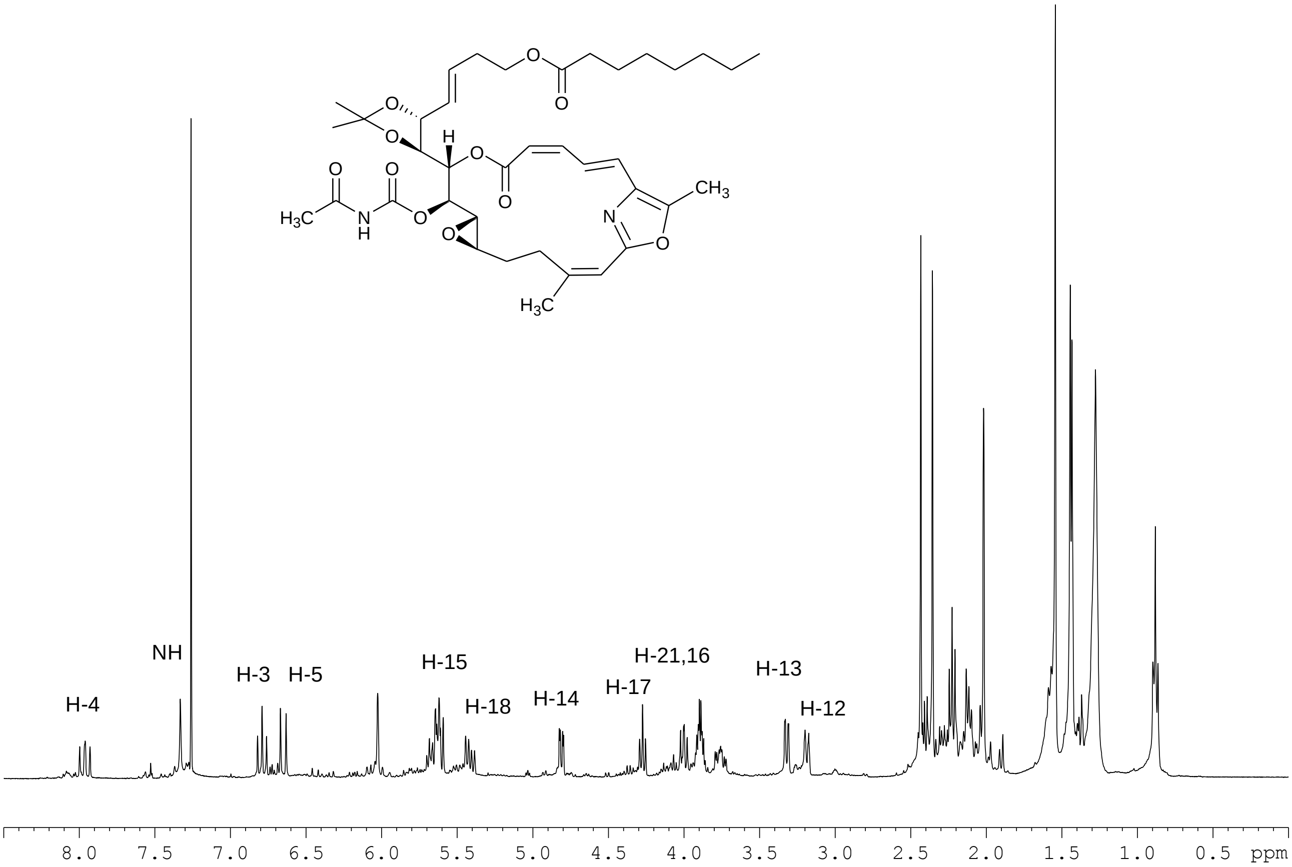

Figure S4. HSQC of compound **6** ($CDCl_3$, 400 MHz).

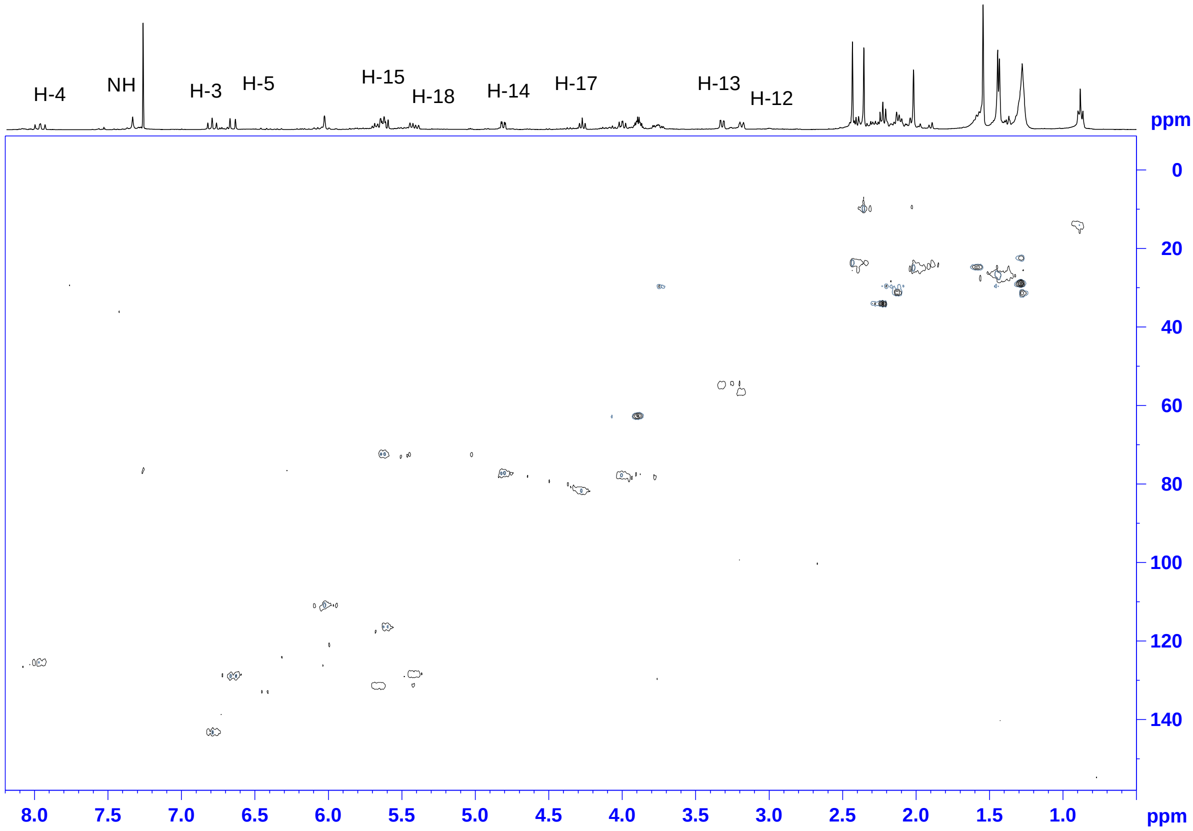

Figure S5. ^{1}H-NMR of compound **7** ($CDCl_3$, 500 MHz).

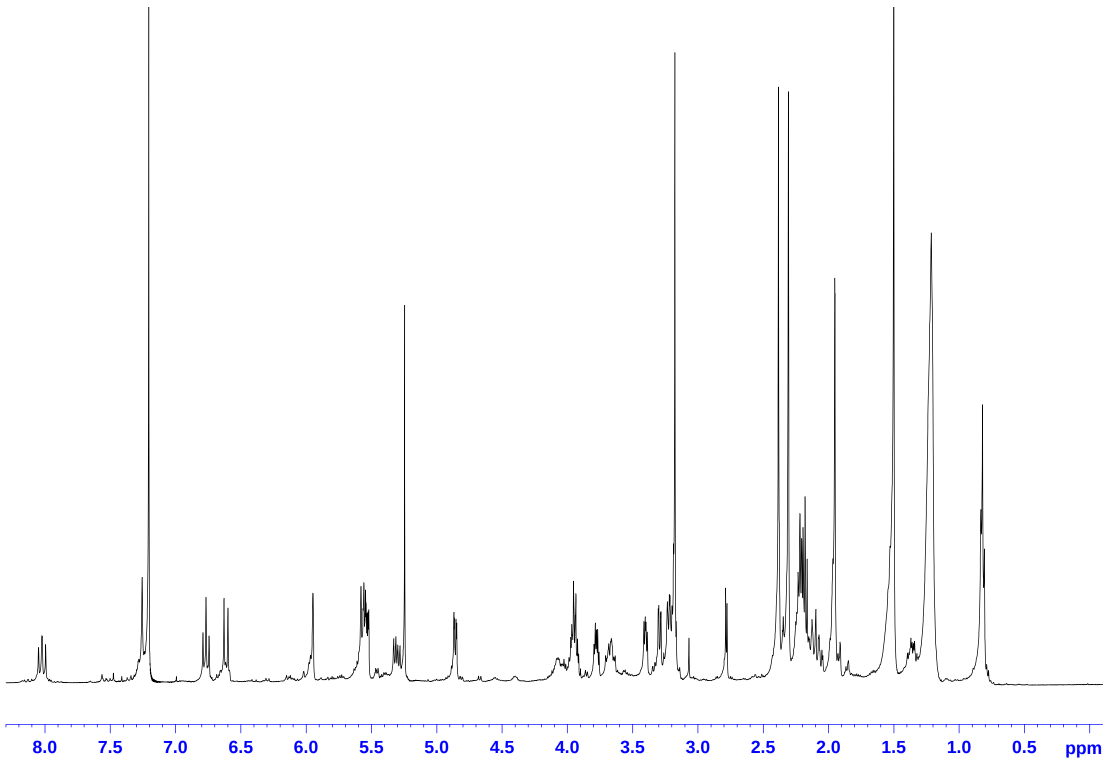

Figure S6. HMBC of compound **7** ($CDCl_3$, 500 MHz).

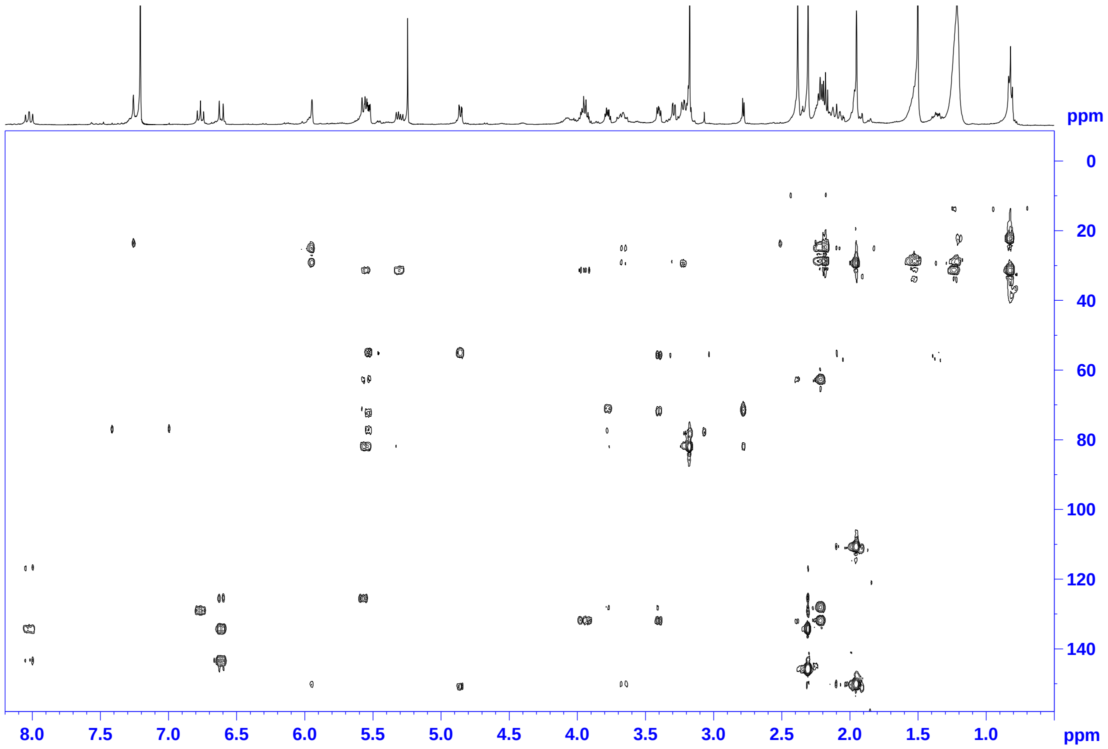

Figure S7. ^{1}H-NMR of compound **9** ($CDCl_3$, 500 MHz).

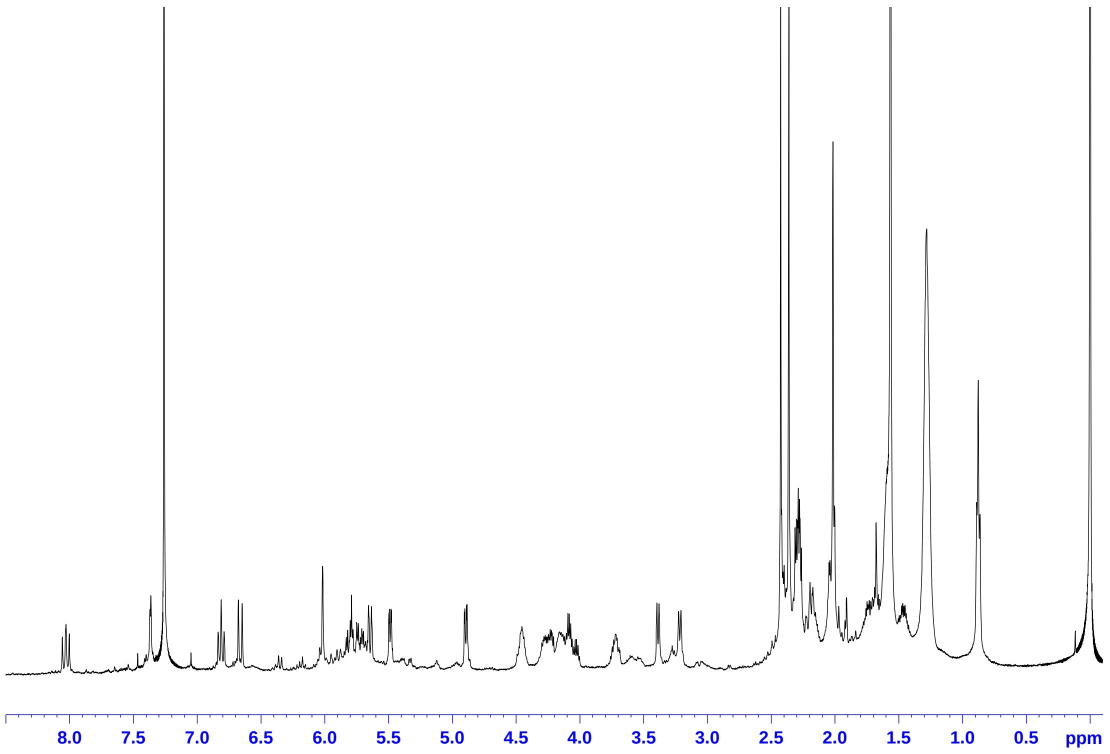

Figure S8. HSQC of compound **9** ($CDCl_3$, 500 MHz).

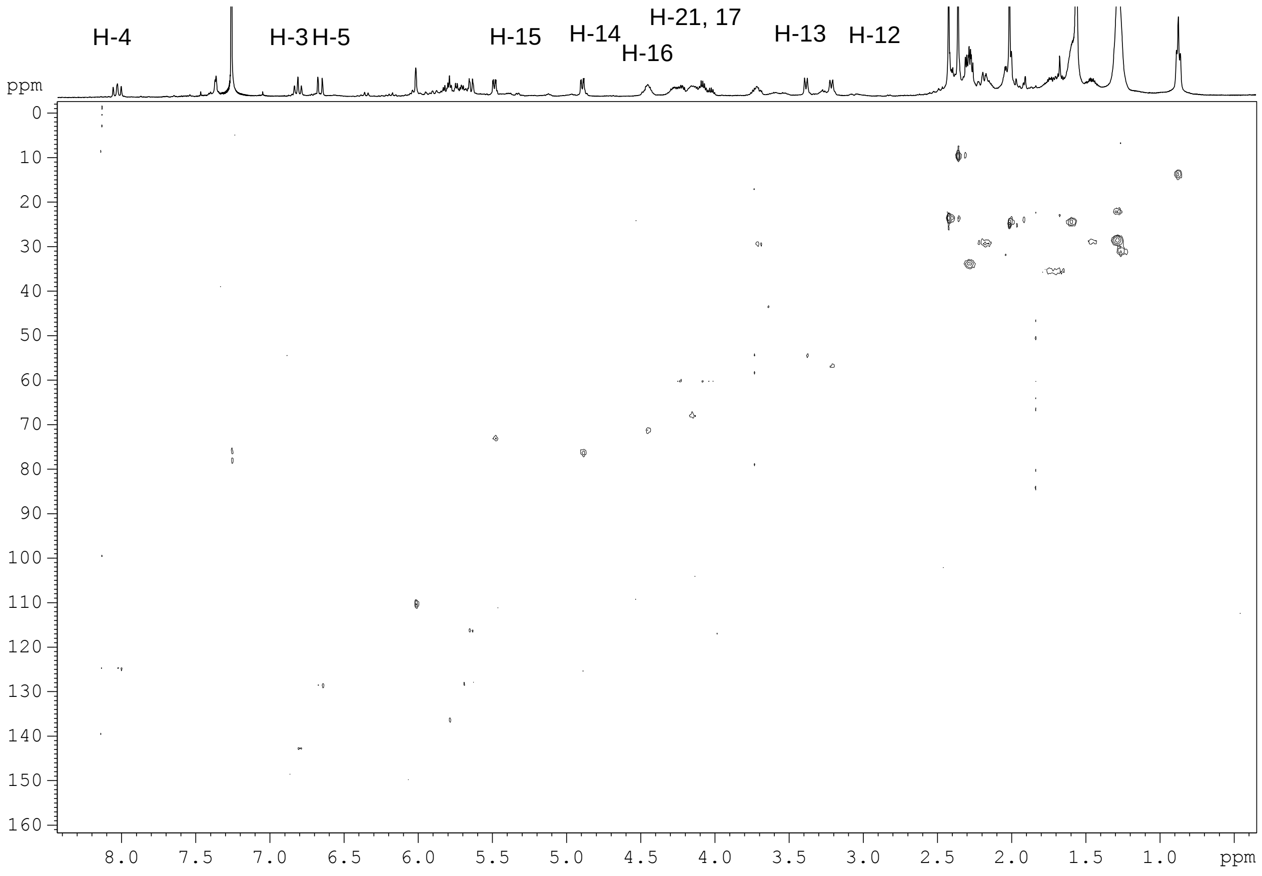

Figure S9. ^{1}H-NMR of compound **11** ($CDCl_3$, 500 MHz).

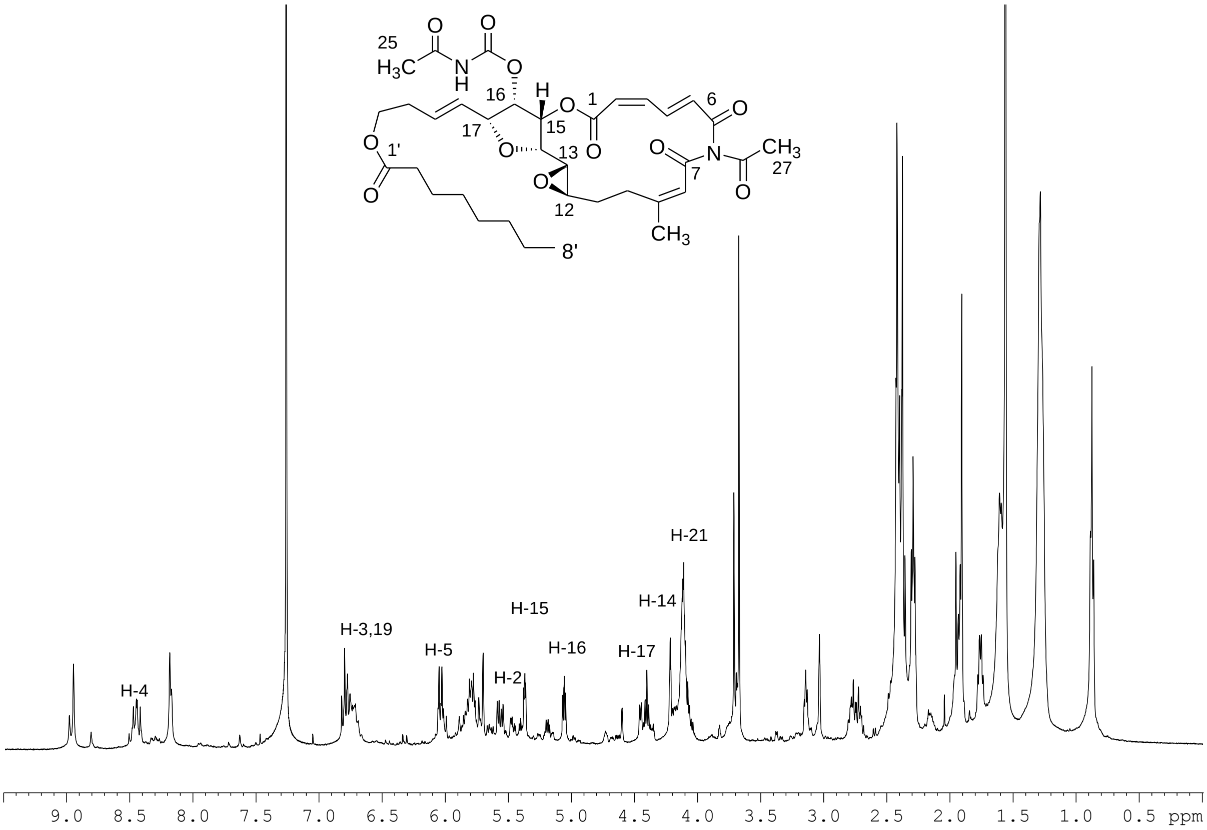

Figure S10. HMBC of compound **11** ($CDCl_3$, 500 MHz).

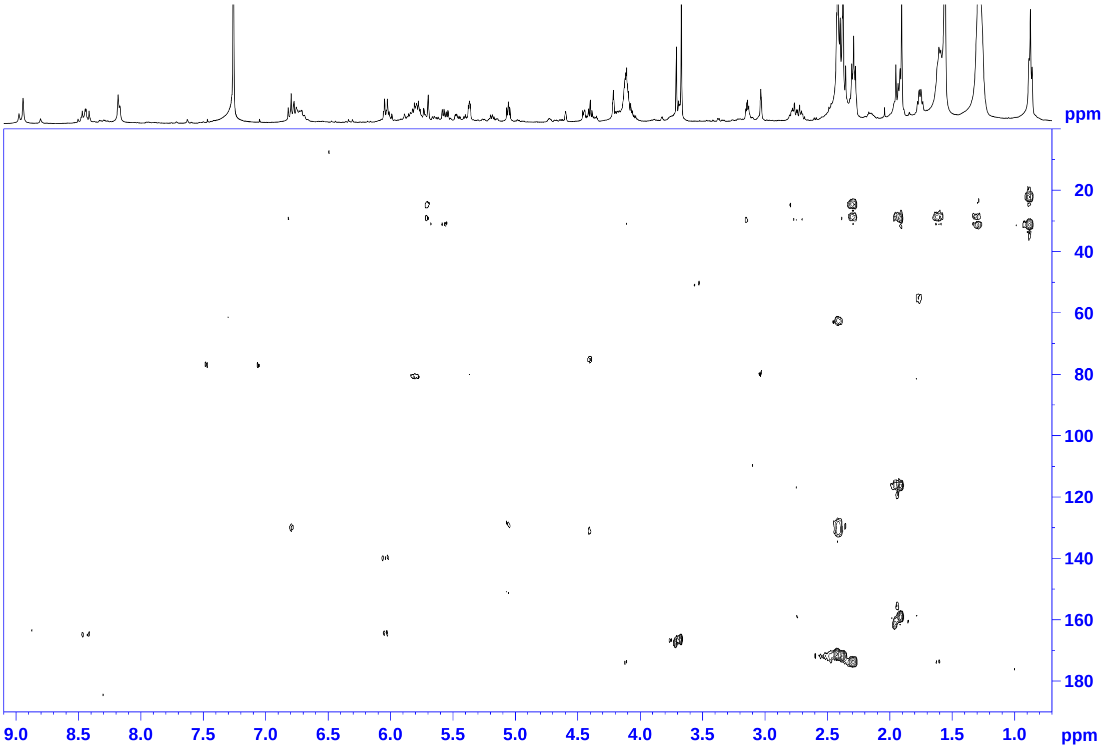

Figure S11. ^{1}H-NMR of compound **13** ($CDCl_3$, 500 MHz).

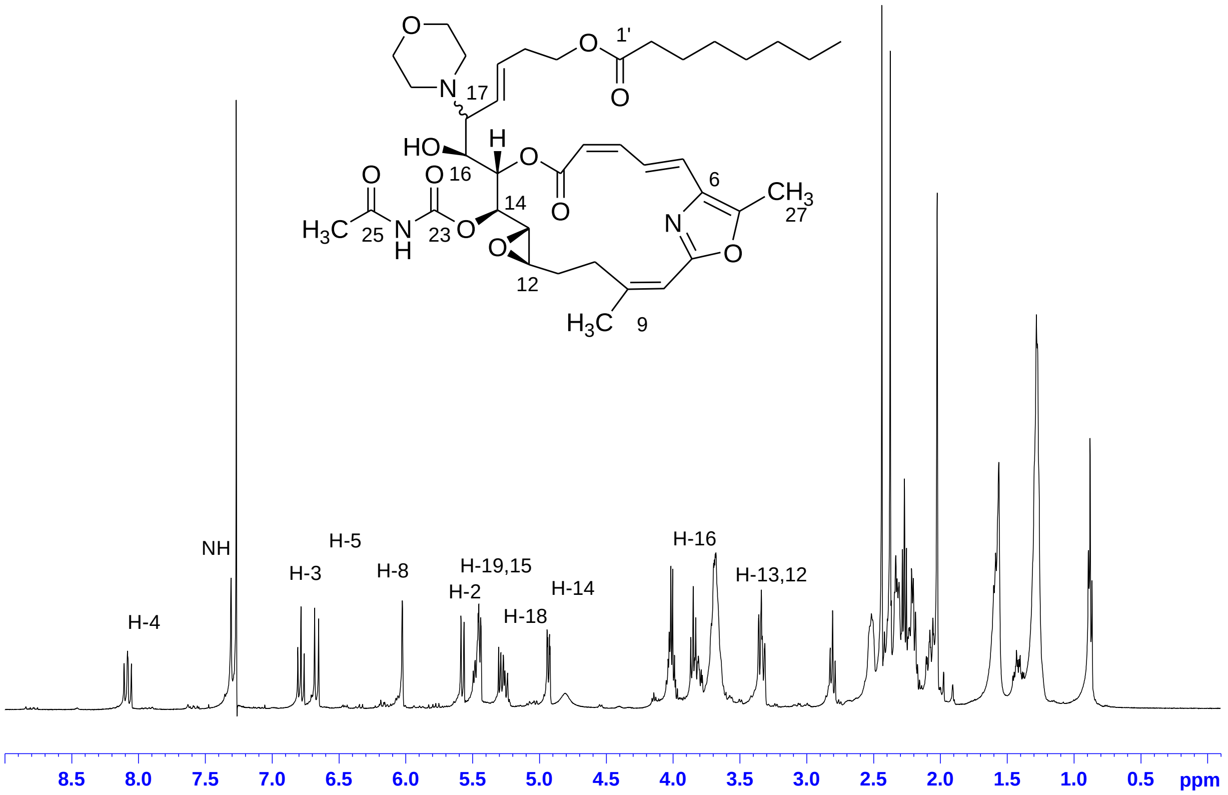

Figure S12. HSQC of compound **13** ($CDCl_3$, 500 MHz).

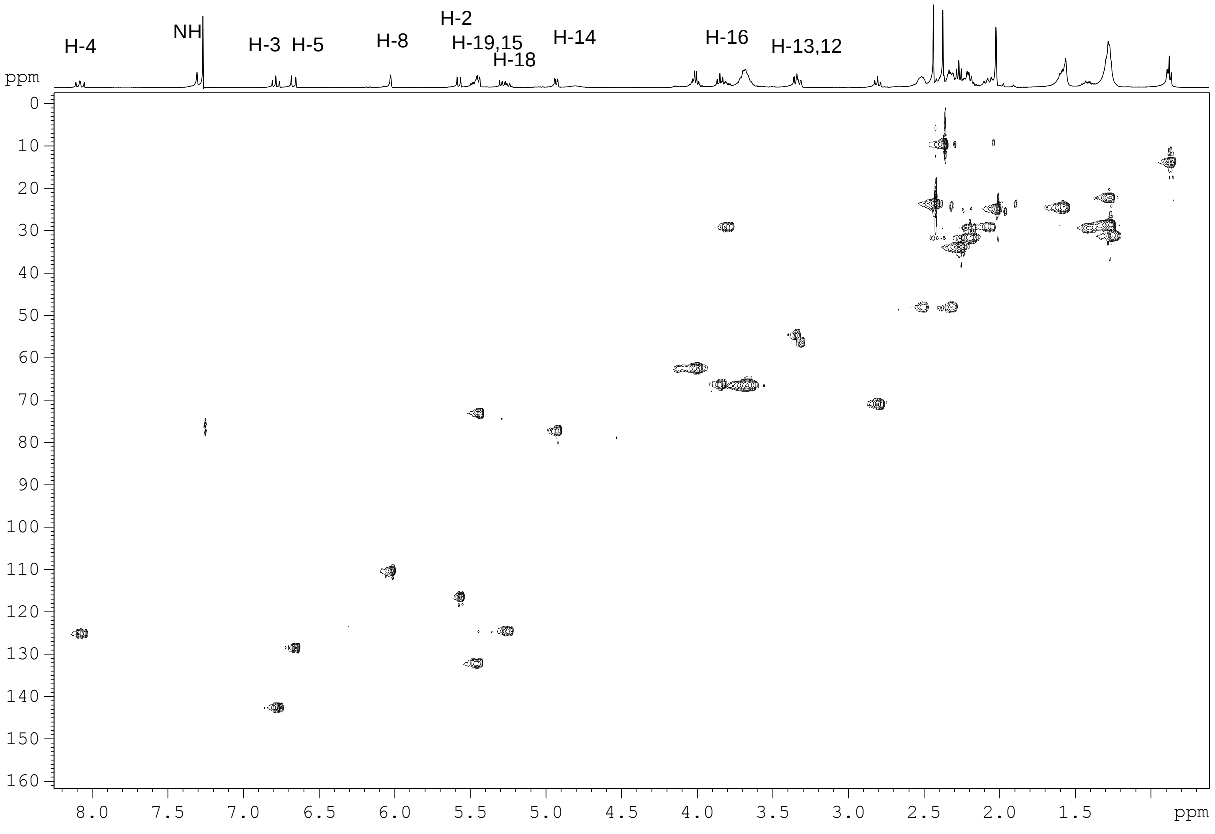

Figure S13. ^{1}H-NMR of compound **19** ($CDCl_3$, 500 MHz).

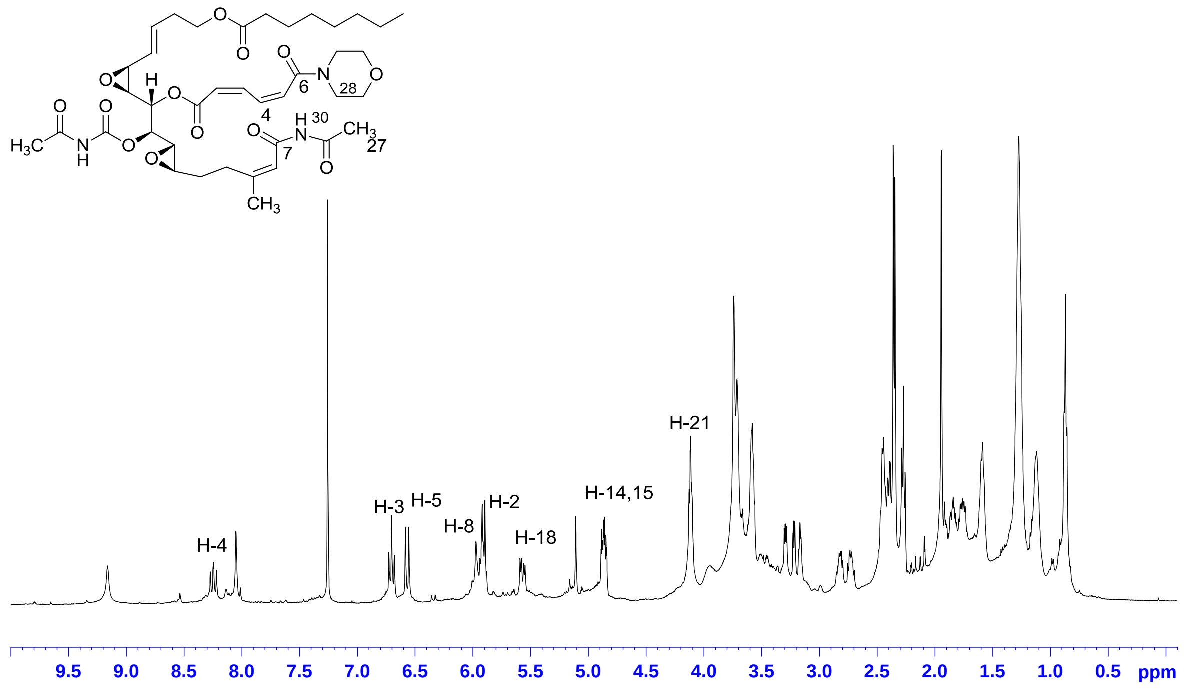

Figure S14. HMBC of compound **19** ($CDCl_3$, 500 MHz).

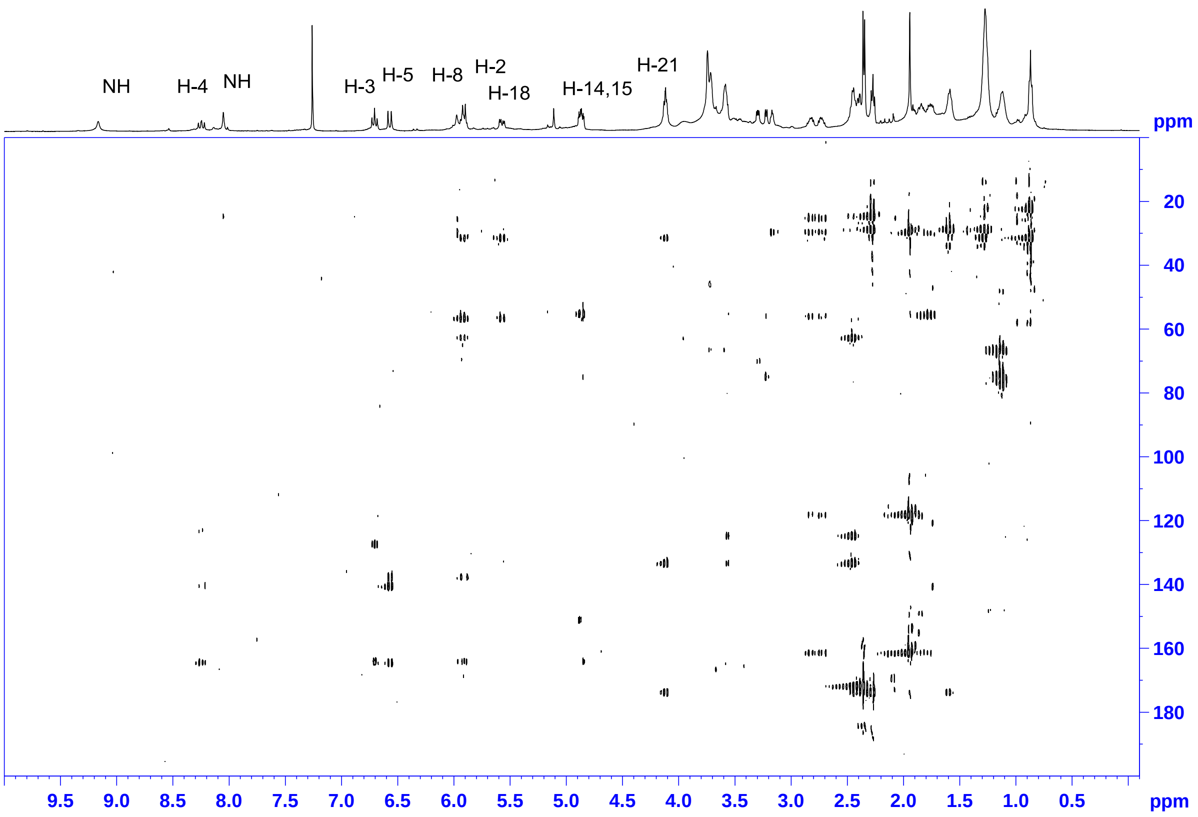

Figure S15. ^{1}H-NMR of compound **21** ($CDCl_3$, 400 MHz).

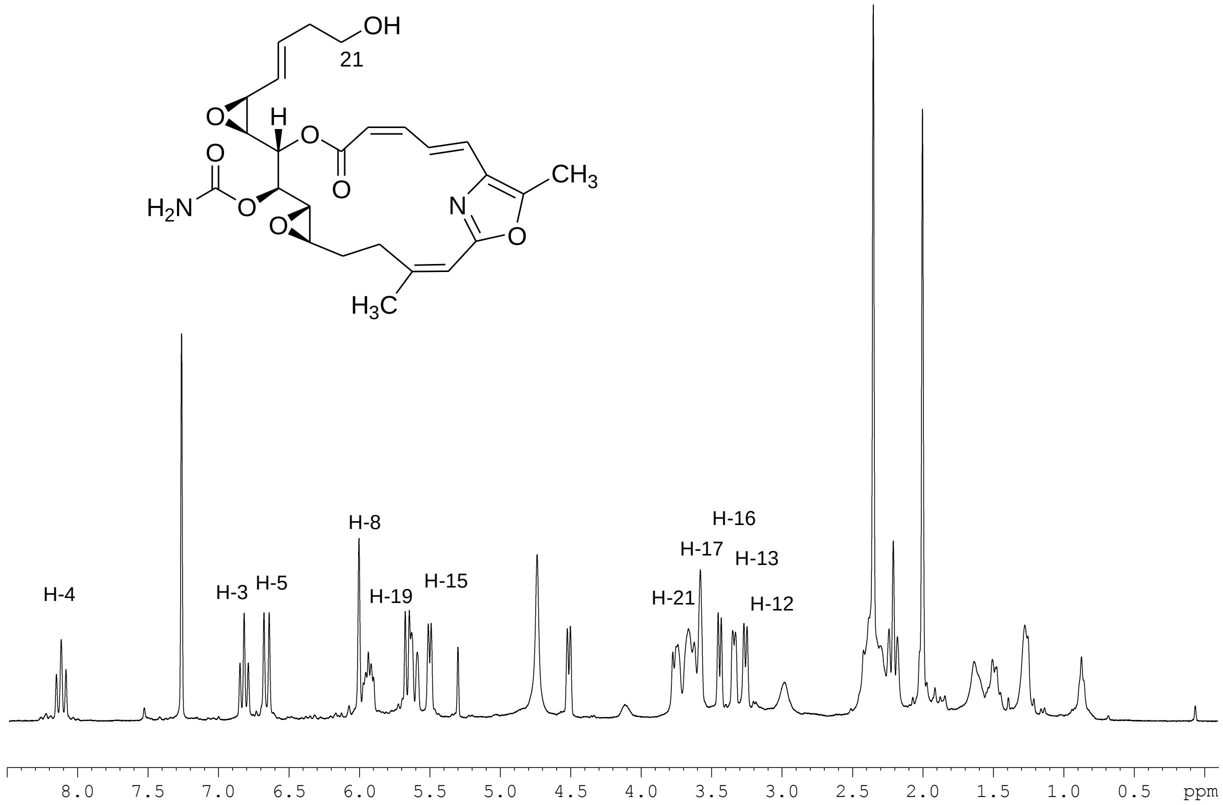

Figure S16. ^{13}C-NMR of compound **21** ($CDCl_3$, 100 MHz).

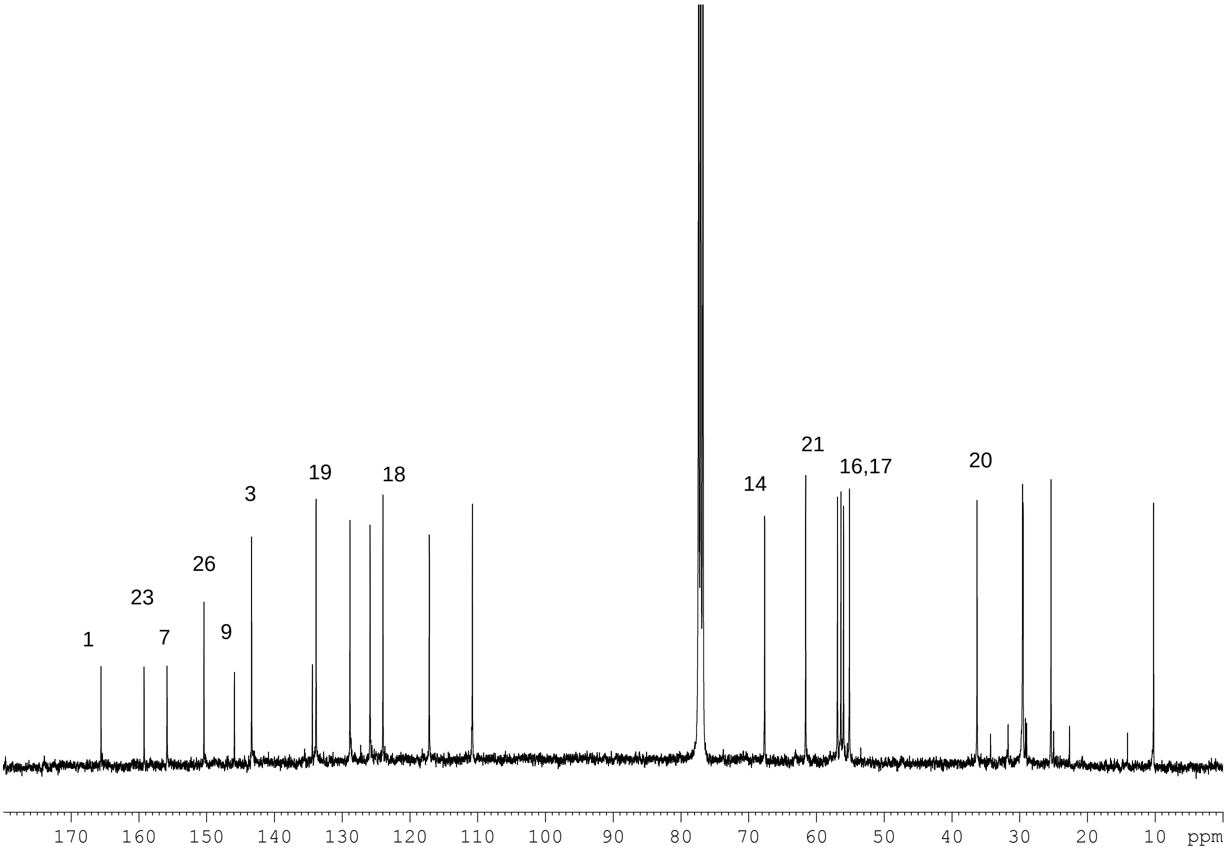

Figure S17. ^{1}H-NMR of compound **24** ($CDCl_3$, 400 MHz).

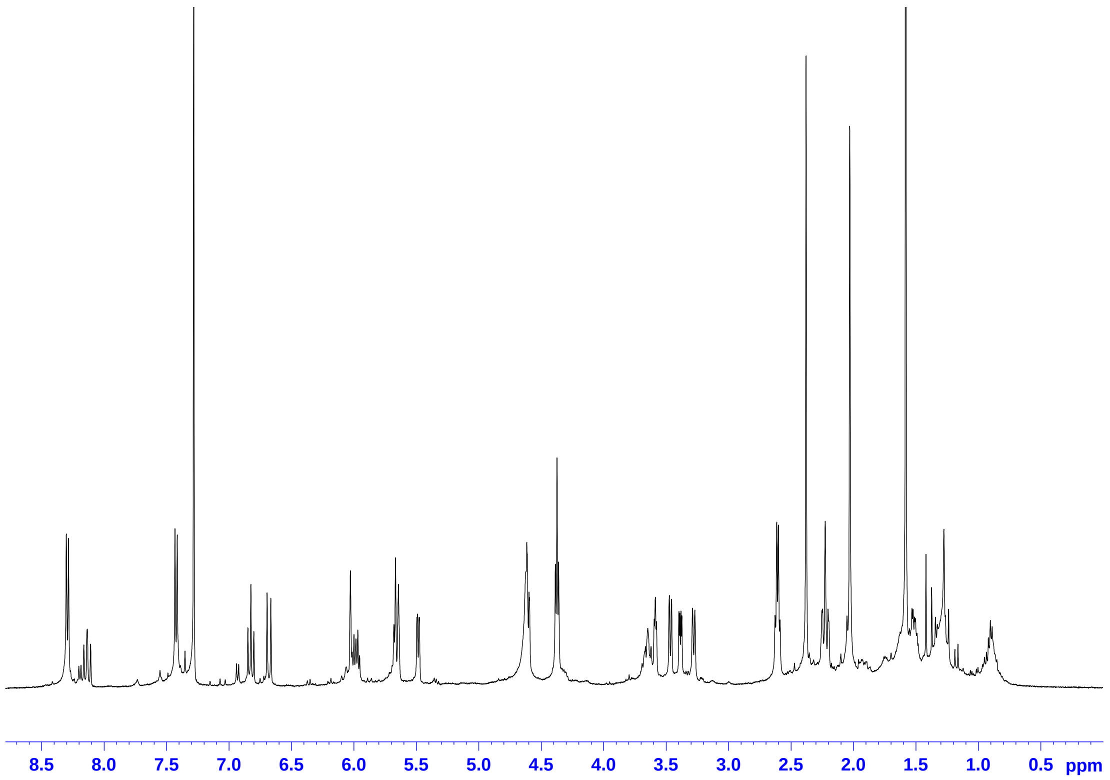

Figure S18. ^{13}C-NMR of compound **24** ($CDCl_3$, 100 MHz).

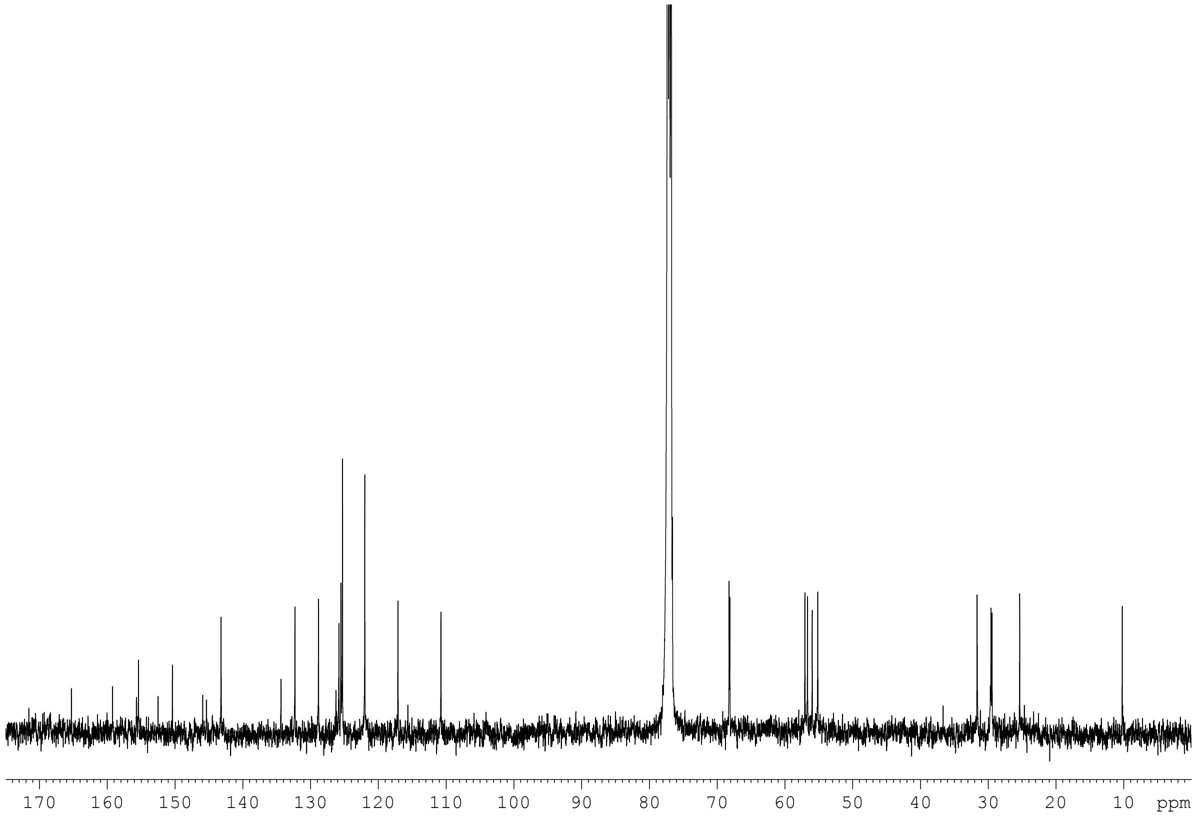

Figure S19. ^{1}H-NMR of compound **25** (d_4-MeOH, 500 MHz).

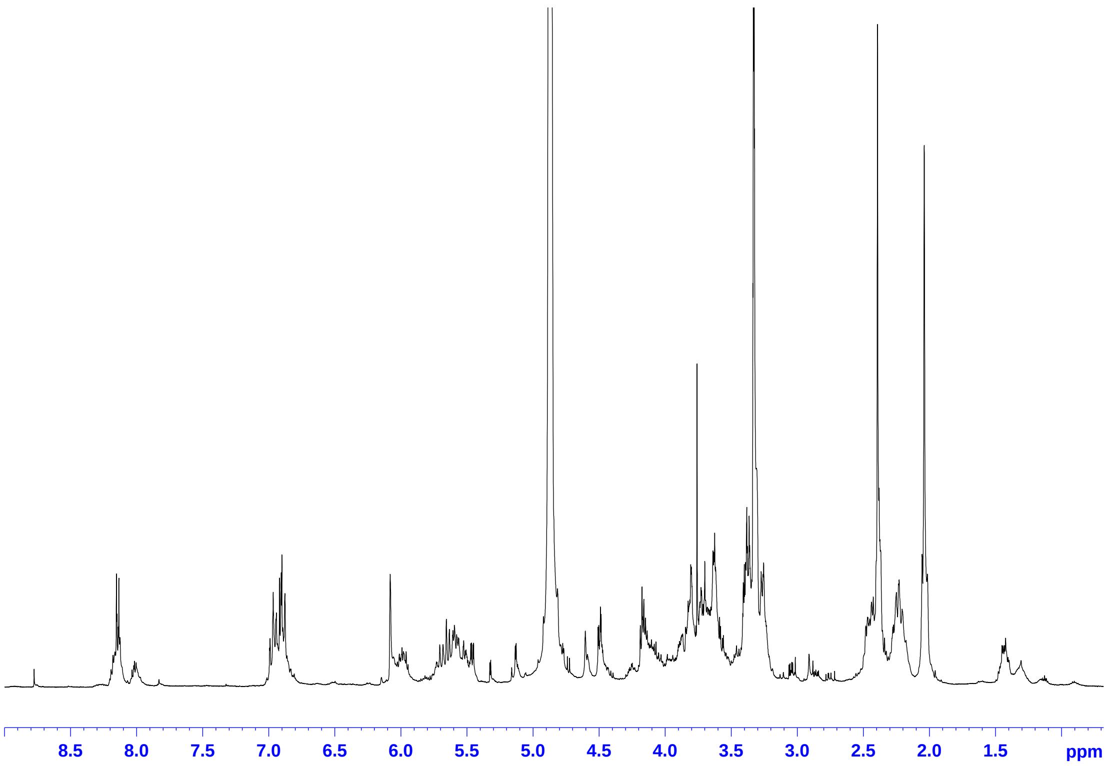

Figure S20. ^{13}C-NMR of compound **25** (d_4-MeOH, 125 MHz).

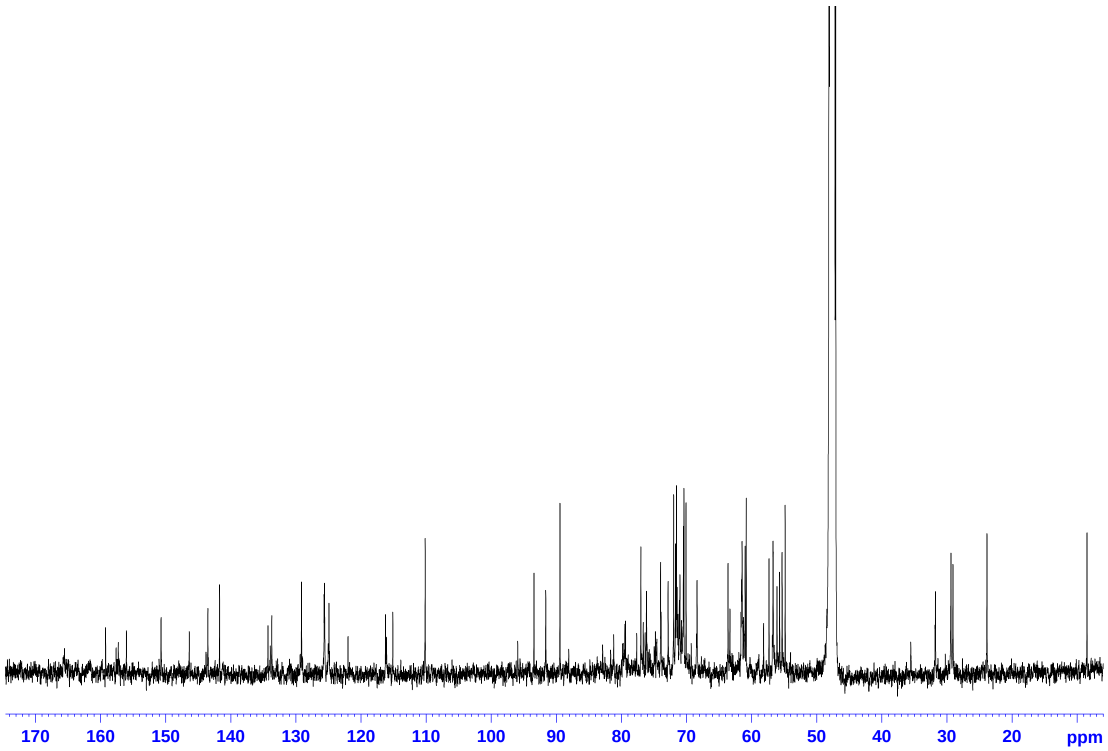

Supplement: Supplementary File 1 — Supporting Information (PDF, 978 KB) [file marinedrugs-11-04487-s001.pdf]
